# Comparative Effectiveness of Empagliflozin vs Liraglutide or Sitagliptin in Older Adults With Diverse Patient Characteristics

**DOI:** 10.1001/jamanetworkopen.2022.37606

**Published:** 2022-10-20

**Authors:** Phyo T. Htoo, Helen Tesfaye, Sebastian Schneeweiss, Deborah J. Wexler, Brendan M. Everett, Robert J. Glynn, Seoyoung C. Kim, Mehdi Najafzadeh, Lisette Koeneman, Soulmaz Fazeli Farsani, Anouk Déruaz-Luyet, Julie M. Paik, Elisabetta Patorno

**Affiliations:** 1 Division of Pharmacoepidemiology and Pharmacoeconomics, Department of Medicine, Brigham and Women’s Hospital and Harvard Medical School, Boston, Massachusetts; 2 Massachusetts General Hospital Diabetes Center, Harvard Medical School, Boston, Massachusetts; 3 Division of Preventive Medicine, Department of Medicine, Brigham and Women’s Hospital, Harvard Medical School, Boston, Massachusetts; 4 Division of Rheumatology, Inflammation, and Immunity, Brigham and Women’s Hospital, Harvard Medical School, Boston, Massachusetts; 5 Global Medical Affairs, Lilly Deutschland, Aalen, Germany; 6 Global Epidemiology, Boehringer Ingelheim International, Ingelheim am Rheim, Germany

## Abstract

**Importance:**

Limited evidence is available on the comparative effectiveness of empagliflozin vs alternative second-line glucose-lowering agents in patients with type 2 diabetes (T2D) receiving routine care who have a broad spectrum of cardiorenal risk.

**Objective:**

To evaluate the association of empagliflozin with cardiovascular outcomes relative to liraglutide and sitagliptin, stratified by age, sex, baseline atherosclerotic cardiovascular disease (ASCVD), heart failure (HF), and chronic kidney disease (CKD).

**Design, Setting, and Participants:**

This retrospective comparative effectiveness cohort study used deidentified Medicare claims data from August 1, 2014, to September 30, 2018, with follow-up from drug initiation until treatment changes, death, or gap in Medicare enrollment (>30 days). Data analysis was performed from October 1, 2021, to April 30, 2022. Medicare fee-for-service beneficiaries older than 65 years with T2D were included. A total of 45 788 patients (22 894 propensity score–matched pairs initiating treatment with either empagliflozin or liraglutide) were included in cohort 1, and 45 624 patients (22 812 propensity score–matched pairs initiating treatment with either empagliflozin or sitagliptin) were included in cohort 2.

**Exposures:**

Empagliflozin vs liraglutide (cohort 1) or empagliflozin vs sitagliptin (cohort 2).

**Main Outcomes and Measures:**

Primary outcomes were (1) modified major adverse cardiovascular events (MACEs), including a composite of myocardial infarction, stroke, and all-cause mortality, and (2) hospitalization for heart failure (HHF). Hazard ratios (HRs) and rate differences (RDs) per 1000 person-years were estimated, adjusting for 143 baseline covariates using 1:1 propensity score matching.

**Results:**

Among 45 788 patients in cohort 1, the mean (SD) age was 71.9 (5.1) years; 23 396 patients (51.1%) were female, 22 392 (48.9%) were male, and 38 049 (83.1%) were White. Among 45 624 patients in cohort 2, the mean (SD) age was 72.1 (5.1) years; 21 418 patients (46.9%) were female, 24 206 (53.1%) were male, and 37 814 (82.9%) were White. Relative to patients initiating liraglutide, those initiating empagliflozin had a similar risk of the modified MACE outcome (HR, 0.90; 95% CI, 0.79-1.03) and a reduced risk of HHF (HR, 0.66; 95% CI, 0.52-0.82). Across subgroups, empagliflozin was associated with a lower risk of the modified MACE outcome in patients with a history of ASCVD (HR, 0.83; 95% CI, 0.71-0.98) and HF (HR, 0.77; 95% CI, 0.60-1.00) compared with liraglutide, and potential heterogeneity in estimates was observed by sex (male: HR, 0.85 [95% CI, 0.71-1.01]; female: HR, 1.16 [95% CI, 0.94-1.42]; *P* = .02 for homogeneity). However, reductions in the risk of HHF were observed across most subgroups (eg, ASCVD: HR, 0.66 [95% CI, 0.51-0.85]; HF: HR, 0.66 [95% CI, 0.49-0.88]). Compared with sitagliptin, empagliflozin was associated with reduced risks of the modified MACE outcome (HR, 0.68; 95% CI, 0.60-0.77) and HHF (HR, 0.45; 95% CI, 0.36-0.56), which were consistent across all subgroups. Absolute benefits of empagliflozin vs sitagliptin were larger in patients with a history of ASCVD (modified MACE: RD, −17.6 [95% CI, −24.9 to −10.4]; HHF: RD, −16.7 [95% CI, −21.7 to −11.9]), HF (modified MACE: RD, −41.1 [95% CI, −59.9 to −22.6]; HHF: RD, −50.4 [95% CI, −67.5 to −33.9]), or CKD (modified MACE: RD, −26.7 [95% CI, −41.3 to −12.3]; HHF: RD, −31.9 [95% CI, −43.5 to −20.8]).

**Conclusions and Relevance:**

In this comparative effectiveness study of older adults, empagliflozin was associated with a lower risk of HHF (relative to both liraglutide and sitagliptin) and the modified MACE outcome (relative to sitagliptin), with larger absolute benefits in patients with established cardiorenal diseases. These findings suggest that older adults with T2D might benefit more from empagliflozin vs liraglutide or sitagliptin with respect to the risk of HHF; with respect to the risk of MACEs, empagliflozin might be preferable to liraglutide only in patients with cardiovascular disease history and to sitagliptin across all patient subgroups.

## Key Points

**Question:**

What is the comparative risk of cardiovascular outcomes associated with empagliflozin vs liraglutide or sitagliptin, overall and across strata of age, sex, baseline atherosclerotic cardiovascular diseases, heart failure, and chronic kidney disease?

**Findings:**

In this comparative effectiveness study of 45 788 patients with type 2 diabetes initiating empagliflozin vs liraglutide and 45 624 patients initiating empagliflozin vs sitagliptin, empagliflozin was associated with a lower risk of hospitalization for heart failure (HHF) vs liraglutide and with both modified major adverse cardiovascular events and HHF vs sitagliptin, with larger absolute benefits in patients with cardiorenal diseases.

**Meaning:**

These findings suggest that older adults with type 2 diabetes might benefit more from empagliflozin vs liraglutide or sitagliptin with respect to the risk of HHF; with respect to the risk of major cardiovascular events, empagliflozin might be preferable to liraglutide only in patients with cardiovascular disease history and to sitagliptin across all patient subgroups.

## Teaser

This comparative effectiveness study evaluates the association of empagliflozin with cardiovascular outcomes compared with liraglutide and sitagliptin among older adults with type 2 diabetes and diverse characteristics.

## Introduction

Older adults with type 2 diabetes (T2D) are at an increased risk of cardiovascular disease (CVD) compared with those without T2D.^1^ Although professional societies recommend the 2 classes of glucose-lowering agents, sodium-glucose cotransporter 2 (SGLT2) inhibitors and glucagon-like peptide-1 receptor agonists (GLP-1RAs), for their cardioprotective effects,^2,3^ adoption of these guidelines in routine care remains a challenge because evidence suggests that their cardiovascular benefits might not be uniform across patient subgroups.

In particular, the protective effects of both SGLT2 inhibitors and GLP-1RAs on major adverse cardiovascular events (MACEs) were more substantial in patients with established atherosclerotic cardiovascular disease (ASCVD) or heart failure (HF), whereas their effects on hospitalization for heart failure (HHF) did not differ by baseline CVD status.^4,5^ Age is also a potential modifier of the cardiovascular effects of SGLT2 inhibitors and GLP-1RAs; for example, empagliflozin, an SGLT2 inhibitor, was found to reduce the risk of cardiovascular death and MACEs only in patients older than 65 years,^6,7^ and liraglutide, the first agent in the GLP-1RA class to have reported cardioprotective benefits, reduced the risk of MACEs to a larger extent in patients older than 75 years compared with those younger than 75 years.^8^

Currently, limited evidence is available^9^ on the direct comparison of empagliflozin with liraglutide (or GLP-1RA class) in nonselected patients with a broader spectrum of ASCVD and/or HF risk and a higher mean age in routine clinical practice than those enrolled in clinical trials.^10^ This evidence across various subgroups of patients in routine care settings could help guide treatment decision-making by tailoring medications to those who will most benefit from them.

We therefore compared the cardiovascular benefits of empagliflozin (the use of which has been increasing in recent years^11^) relative to (1) liraglutide, the most frequently used GLP-1RA with demonstrated cardiovascular benefits,^12^ and (2) sitagliptin, the most frequently used agent within another glucose-lowering medication class (dipeptidyl peptidase-4 [DPP-4] inhibitors), with a demonstrated neutral cardiovascular effect, both overall and within subgroups of patients with cardiorenal diseases.^13^

## Methods

We conducted a new-user comparative-effectiveness cohort study using the Medicare fee-for-service claims database. This database contains information on patient demographic characteristics and the longitudinal history of reimbursed medical services, including diagnoses, procedures, and pharmacy drug-dispensing records for eligible patients older than 65 years. The study protocol was approved by the institutional review board of Mass General Brigham. Informed consent was waived because the database used in this study contains only deidentified patient records. This study followed the International Society for Pharmacoeconomics and Outcomes Research (ISPOR) reporting guideline for comparative effectiveness studies.

### Study Population

Our study population included 2 pairwise comparison groups of older adults (aged >65 years) who initiated (1) empagliflozin vs liraglutide (cohort 1) and (2) empagliflozin vs sitagliptin (cohort 2) between August 1, 2014 (date of the first approval of empagliflozin in the US), and September 30, 2018. Data analysis was performed from October 1, 2021, to April 30, 2022. For each cohort, cohort entry was the date of the first filled prescription of any of the study drugs after 12 months of no prescription fills for any SGLT2 inhibitor and its respective comparator class (GLP-1RAs for cohort 1 and DPP-4 inhibitors for cohort 2). Because eligibility for empagliflozin initiators was comparator specific (ie, eligible patients who initiated empagliflozin in cohort 1 could not use a GLP-1RA before cohort entry, whereas those in cohort 2 could not use a DPP-4 inhibitor before cohort entry), the numbers of patients initiating empagliflozin who were entering cohorts 1 and 2 were different. We restricted the study population to participants with continuous enrollment in Medicare Part A (inpatient services), Part B (outpatient and physician services), and Part D (prescription medications) insurance plans during the 12-month period before drug initiation, which was defined as a baseline period during which all eligibility criteria were assessed.

Patients were required to have at least 1 diagnosis of T2D recorded in any care setting. We excluded patients with recorded diagnoses of type 1 or secondary diabetes, cancer, end-stage kidney disease or receipt of kidney replacement therapy, or HIV and those who received a solid organ transplant or had a nursing home admission at baseline. To ensure similar drug indications between treatment comparisons, we excluded those with a baseline prescription for liraglutide indicated for weight loss (Saxenda; Novo Nordisk) from cohort 1.

We followed patients from 1 day after cohort entry until the earliest occurrence of the following events: discontinuation of the index drug, switching to the comparator drug, switching to an alternative agent within the same drug class (eg, empagliflozin to dapagliflozin or liraglutide to semaglutide), gap in Medicare enrollment (>30 days), death, end of the study period (September 30, 2018), or occurrence of the study outcomes. We considered patients to be exposed to the index drug until 60 days after the end of the days’ supply of the last prescription.

### Outcomes

Primary outcomes included (1) modified MACE, defined as a composite of myocardial infarction (MI), ischemic or hemorrhagic stroke, and all-cause mortality, and (2) HHF based on HF diagnosis recorded in the first diagnosis field on hospital discharge (HHF specific). Secondary outcomes included individual components of the modified MACE and HHF diagnosis in any discharge position (HHF broad).

We identified outcomes using diagnosis codes from the *International Classification of Diseases, Ninth Revision, Clinical Modification* (*ICD-9-CM*) or the *International Classification of Diseases, Tenth Revision, Clinical Modification* (*ICD-10-CM*) in inpatient settings following Medicare claims–based algorithms from previous validation studies,^14,15,16,17,18^ which reported high specificity (93%-98%) and positive predictive value (>98%). Date of death was ascertained through the Vital Status files in the Medicare database, with approximately 100% of dates of death having been validated against death certificate data.^19^

### Potential Confounders and Baseline Subgroups

Based on the clinical knowledge and the literature review, we identified 143 baseline covariates, including patient demographic characteristics (age, sex, and race and ethnicity, as identified from Medicare enrollment files), US Census region, calendar time of cohort entry, diabetes complications, CVDs, systemic comorbidities, modified Charlson and Elixhauser combined comorbidity scores,^20^ use of chronic disease medications, validated claims-based frailty index scores,^21^ and measures of health care use as a proxy for the intensity of care. We defined these covariates using the claims-based diagnosis and procedure codes or National Drug Codes.

Analyses were stratified by the following subgroups: (1) age (<75 years vs ≥75 years on the cohort entry date), (2) sex (male vs female), (3) baseline history of ASCVD (defined as a diagnosis or procedure code for any of the following conditions: MI, angina, coronary atherosclerosis or other forms of chronic ischemic heart disease, coronary procedure, ischemic stroke, peripheral arterial disease or surgical procedure, or lower extremity amputation) vs no baseline history of ASCVD, (4) presence vs absence of baseline HF, and (5) presence vs absence of baseline chronic kidney disease (CKD).

### Statistical Analysis

We estimated the probability (ie, the propensity score [PS]) of initiating empagliflozin vs each comparator, conditional on the covariates, using multivariable logistic regression analysis.^22^ To control for confounding, we matched patients who initiated empagliflozin with patients who initiated liraglutide or sitagliptin on a 1:1 ratio using the PS based on the nearest-neighbor matching algorithm without replacement, with a specified maximum caliper of 0.01 on the PS scale.^23^ For the prespecified subgroup analyses, we estimated and matched PS separately within each subgroup. We assessed covariate balance between empagliflozin and the matched comparator medications using absolute standardized mean differences^24^ and the postmatched C statistic of the model estimating empagliflozin vs comparator medications, conditional on the baseline covariates (with 0.5 indicating satisfactory balance).^25^

Treatment effect was estimated using hazard ratios (HRs) from a Fine-Gray Cox regression model and absolute rate differences (RDs) using weighted least squares regression models.^26^ The heterogeneity of treatment effect across subgroups was detected using the Wald test for homogeneity. We also assessed the risk of outcome over the follow-up period using cumulative incidence function plots, accounting for the competing risk of death.^27^

To address potential exposure misclassification, we varied the exposure assessment window from 60 days to 30 days before censoring for treatment discontinuation or drug switching. We addressed potential informative censoring by conducting intention-to-treat analyses without censoring for treatment discontinuation or switching, following up patients until 2 years after cohort entry. To evaluate the presence of potential residual confounding, we (1) restricted the cohort to those having a metformin prescription claim during baseline, (2) excluded patients with insulin prescriptions at baseline, and (3) conducted bias analyses evaluating the estimates obtained after hypothetically adjusting for a strong unmeasured confounder.^28^ As secondary analyses, we compared empagliflozin vs the entire drug classes of GLP-1RAs or DPP-4 inhibitors.

All analyses were performed using the Aetion Evidence Platform (2021; Aetion Inc), with R software, version 4.1 (R Foundation for Statistical Computing),^29^ and SAS statistical software, version 9.4 (SAS Institute Inc). The significance threshold for the Wald test for homogeneity was 2-sided *P* = .05.

## Results

### Cohort Characteristics

Before PS matching, the study populations included 85 121 patients in cohort 1 (35 721 patients who initiated empagliflozin vs 49 400 patients who initiated liraglutide) and 224 178 patients in cohort 2 (25 285 patients who initiated empagliflozin vs 198 893 patients who initiated sitagliptin) (eFigure 1 and eFigure 2 in the Supplement). After 1:1 PS matching, we identified 45 788 patients in cohort 1 (22 894 matched pairs) and 45 624 patients in cohort 2 (22 812 matched pairs).

Before PS matching, when compared with patients who initiated liraglutide, those who initiated empagliflozin had similar distributions of age (mean [SD], 72.4 [5.3] years vs 71.4 [4.9] years) and cardiovascular comorbidities (mean [SD] combined comorbidity score, 1.4 [2.0] vs 1.7 [2.2]), with a higher proportion of baseline metformin use (28 427 patients [79.6%] vs 34 165 patients [69.2%]) and a lower proportion of baseline insulin use (8401 patients [23.5%] vs 21 635 patients [43.8%]) (eTable 1 in the Supplement). Relative to patients who initiated sitagliptin, before PS matching, those who initiated empagliflozin were younger (mean [SD], 71.9 [5.0] years vs 74.2 [6.6] years) and less likely to be female (11 556 patients [45.7%] vs 111 998 patients [56.3%]) or Black or African American (1649 patients [6.5%] vs 20 936 patients [10.5%]), with a higher proportion of baseline insulin use (7982 patients [31.6%] vs 30 931 patients [15.6%]).

After 1:1 PS matching, among 45 788 participants in cohort 1 (empagliflozin vs liraglutide), the mean (SD) age was 71.9 (5.1) years; 23 396 patients (51.1%) were female, 22 392 (48.9%) were male, 1094 (2.4%) were Asian, 3303 (7.2%) were Black or African American, 1183 (2.6%) were Hispanic, 38 049 (83.1%) were White, and 2159 (4.7%) were of unknown or other race and/or ethnicity (including American Indian or Alaska Native) (Table 1). Among 45 624 participants in cohort 2 (empagliflozin vs sitagliptin), the mean (SD) age was 72.1 (5.1) years; 21 418 patients (46.9%) were female, 24 206 (53.1%) were male, 1236 (2.7%) were Asian, 3098 (6.8%) were Black or African American, 1209 (2.6%) were Hispanic, 37 814 (82.9%) were White, and 2267 (5.0%) were of unknown or other race and/or ethnicity (including American Indian or Alaska Native). Baseline characteristics were similar between treatment groups in both cohort 1 (eg, 22 894 patients initiating empagliflozin vs 22 894 initiating liraglutide: mean [SD] combined comorbidity score, 1.50 [2.03] vs 1.50 [1.99]; CKD stage 3-4, 3066 patients [13.4%] vs 3088 patients [13.5%]; long-term insulin use, 4926 patients [21.5%] vs 4857 patients [21.2%]) and cohort 2 (eg, 22 812 patients initiating empagliflozin vs 22 812 initiating sitagliptin: mean [SD] combined comorbidity score, 1.40 [1.96] vs 1.40 [1.95]; CKD stage 3-4, 2376 patients [10.4%] vs 2325 patients [10.2%]; long-term insulin use: 4355 patients [19.1%] vs 4295 patients [18.8%]). The postmatched C statistic of the treatment model conditional on baseline covariates was approximately 0.5, indicating satisfactory balance.

**Table 1.** Characteristics of 1:1 Propensity Score–Matched Participants Initiating Treatment With Empagliflozin vs Liraglutide or Sitagliptin

Characteristic^a^	Participants, No. (%)					
	Empagliflozin vs liraglutide			Empagliflozin vs sitagliptin		
	Empagliflozin (n = 22 894)	Liraglutide (n = 22 894)	ASMD^b^	Empagliflozin (n = 22 812)	Sitagliptin (n = 22 812)	ASMD^b^
Demographic factors						
Age, mean (SD), y	71.9 (5.1)	71.9 (5.1)	0.001	72.1 (5.1)	72.1 (5.1)	0.002
Sex						
Female	11 711 (51.2)	11 685 (51.0)	0.002	10 697 (46.9)	10 721 (47.0)	0.002
Male	11 183 (48.8)	11 209 (49.0)	0.002	12 115 (53.1)	12 091 (53.0)	0.002
Race and ethnicity						
Asian	546 (2.4)	548 (2.4)	0.001	648 (2.8)	588 (2.6)	0.016
Black or African American	1636 (7.1)	1667 (7.3)	0.005	1562 (6.8)	1536 (6.7)	0.005
Hispanic	585 (2.6)	598 (2.6)	0.004	602 (2.6)	607 (2.7)	0.001
White	19 059 (83.2)	18 990 (82.9)	0.008	18 896 (82.8)	18 918 (82.9)	0.003
Other or unknown^c^	1068 (4.7)	1091 (4.8)	0.005	1104 (4.8)	1163 (5.1)	0.012
Burden of comorbidities						
Combined Charlson and Elixhauser comorbidity score, mean (SD)	1.50 (2.03)	1.50 (1.99)	0.002	1.40 (1.96)	1.40 (1.95)	0.001
Frailty index score, mean (SD)	0.20 (0.06)	0.20 (0.05)	0.002	0.20 (0.05)	0.20 (0.05)	0.011
Lifestyle factors						
Overweight^d^	2255 (9.8)	2255 (9.8)	0	2425 (10.6)	2449 (10.7)	0.003
Obesity^d^	9357 (40.9)	9360 (40.9)	0	8616 (37.8)	8606 (37.7)	0.001
Smoking	5248 (22.9)	5253 (22.9)	0.001	5309 (23.3)	5338 (23.4)	0.003
Diabetes-related conditions						
Nephropathy	3945 (17.2)	3993 (17.4)	0.006	3463 (15.2)	3460 (15.2)	0
Retinopathy	3374 (14.7)	3326 (14.5)	0.006	3168 (13.9)	3221 (14.1)	0.007
Neuropathy	6819 (29.8)	6819 (29.8)	0	6394 (28.0)	6475 (28.4)	0.008
Lower-limb amputation	158 (0.7)	141 (0.6)	0.009	141 (0.6)	147 (0.6)	0.003
Hypoglycemia	2411 (10.5)	2363 (10.3)	0.007	2383 (10.4)	2366 (10.4)	0.002
Hyperglycemia	11 331 (49.5)	11 263 (49.2)	0.006	11 040 (48.4)	10 974 (48.1)	0.006
Ketoacidosis	70 (0.3)	78 (0.3)	0.006	61 (0.3)	61 (0.3)	0
Hyperosmolar hyperglycemic nonketosis	231 (1.0)	237 (1.0)	0.003	230 (1.0)	239 (1.0)	0.004
Diabetes treatment						
No. of antidiabetes medications at cohort entry, mean (SD)	1.50 (0.96)	1.50 (0.97)	0.004	1.30 (0.86)	1.30 (0.85)	0.003
Initiation of empagliflozin or comparator monotherapy	895 (3.9)	852 (3.7)	0.010	1038 (4.6)	1050 (4.6)	0.003
Concurrent metformin^e^	13 550 (59.2)	13 612 (59.5)	0.006	14 485 (63.5)	14 504 (63.6)	0.002
Concurrent second-generation sulfonylurea^e^	7978 (34.8)	7965 (34.8)	0.001	7592 (33.3)	7621 (33.4)	0.003
Concurrent thiazolidinedione^e^	1637 (7.2)	1655 (7.2)	0.003	1638 (7.2)	1682 (7.4)	0.007
Concurrent GLP-1RA^e^	5432 (23.7)	5445 (23.8)	0.001	1580 (6.9)	1503 (6.6)	0.013
Concurrent insulin^e^	5675 (24.8)	5681 (24.8)	0.001	4732 (20.7)	4666 (20.5)	0.007
Long-term insulin	4926 (21.5)	4857 (21.2)	0.007	4355 (19.1)	4295 (18.8)	0.007
Other comorbidities						
Acute MI	584 (2.6)	612 (2.7)	0.008	626 (2.7)	605 (2.7)	0.006
MI sequelae or previous MI	1433 (6.3)	1404 (6.1)	0.005	1550 (6.8)	1506 (6.6)	0.008
Unstable angina	881 (3.8)	828 (3.6)	0.012	908 (4.0)	896 (3.9)	0.003
Coronary atherosclerosis	8031 (35.1)	8038 (35.1)	0.001	8370 (36.7)	8316 (36.5)	0.005
Coronary procedure	625 (2.7)	634 (2.8)	0.002	769 (3.4)	781 (3.4)	0.003
Heart failure	3107 (13.6)	3052 (13.3)	0.007	2985 (13.1)	2949 (12.9)	0.005
Cardiomyopathy	1060 (4.6)	1047 (4.6)	0.003	1078 (4.7)	1068 (4.7)	0.002
Atrial fibrillation	2997 (13.1)	3001 (13.1)	0.001	3120 (13.7)	3132 (13.7)	0.002
Ischemic stroke	2858 (12.5)	2847 (12.4)	0.001	2852 (12.5)	2851 (12.5)	0
Peripheral arterial disease	3219 (14.1)	3210 (14.0)	0.001	3043 (13.3)	3058 (13.4)	0.002
Hypertension	21 044 (91.9)	21 075 (92.1)	0.005	20 900 (91.6)	20 898 (91.6)	0
Hyperlipidemia	19 889 (86.9)	19 881 (86.8)	0.001	19 681 (86.3)	19 681 (86.3)	0
CKD stage 3-4	3066 (13.4)	3088 (13.5)	0.003	2376 (10.4)	2325 (10.2)	0.007
Proteinuria	1461 (6.4)	1460 (6.4)	0	1355 (5.9)	1361 (6.0)	0.001
COPD	3018 (13.2)	2988 (13.1)	0.004	2892 (12.7)	2888 (12.7)	0.001
Obstructive sleep apnea	4688 (20.5)	4649 (20.3)	0.004	4347 (19.1)	4279 (18.8)	0.008
Nonalcoholic steatohepatitis or fatty liver disease	1341 (5.9)	1341 (5.9)	0	1297 (5.7)	1274 (5.6)	0.004
Dementia	976 (4.3)	991 (4.3)	0.003	973 (4.3)	957 (4.2)	0.003
Other medications						
ACEI or ARB	18 244 (79.7)	18 284 (79.9)	0.004	17 945 (78.7)	17 904 (78.5)	0.004
β-Blocker	11 418 (49.9)	11 508 (50.3)	0.008	11 427 (50.1)	11 432 (50.1)	0
Calcium channel blocker	7782 (34.0)	7782 (34.0)	0	7681 (33.7)	7588 (33.3)	0.009
Nitrate or other antianginal agent	2335 (10.2)	2352 (10.3)	0.002	2347 (10.3)	2225 (9.8)	0.018
Thiazide	3819 (16.7)	3829 (16.7)	0.001	3694 (16.2)	3638 (15.9)	0.007
Loop diuretic	4541 (19.8)	4521 (19.7)	0.002	4119 (18.1)	4049 (17.7)	0.008
Potassium-sparing diuretic	1271 (5.6)	1226 (5.4)	0.009	1197 (5.2)	1179 (5.2)	0.004
Digoxin	543 (2.4)	538 (2.3)	0.001	571 (2.5)	550 (2.4)	0.006
Antiarrhythmic	612 (2.7)	603 (2.6)	0.002	606 (2.7)	613 (2.7)	0.002
Anticoagulant	2460 (10.7)	2451 (10.7)	0.001	2480 (10.9)	2481 (10.9)	0
Statin	18 328 (80.1)	18 234 (79.6)	0.010	18 290 (80.2)	18 212 (79.8)	0.009
PCSK9 inhibitor or other lipid-lowering agent	4226 (18.5)	4211 (18.4)	0.002	4056 (17.8)	4084 (17.9)	0.003
Oral corticosteroid	4252 (18.6)	4240 (18.5)	0.001	4014 (17.6)	4005 (17.6)	0.001
Opioid	8106 (35.4)	8098 (35.4)	0.001	7630 (33.4)	7631 (33.5)	0
Measures of health care use						
Visit to internist <30 d before cohort entry	14 076 (61.5)	13 931 (60.9)	0.013	14 308 (62.7)	14 391 (63.1)	0.008
Visit to cardiologist <30 d before cohort entry	3387 (14.8)	3396 (14.8)	0.001	3871 (17.0)	3761 (16.5)	0.013
No. of HbA_1c_ tests ordered, mean (SD)	2.90 (1.32)	2.90 (1.34)	0	2.80 (1.30)	2.80 (1.33)	0.004
No. of glucose tests and monitoring ordered, mean (SD)	0.90 (1.85)	0.90 (1.84)	0.002	0.80 (1.81)	0.80 (1.73)	0.007
No. of microalbuminuria/proteinuria tests ordered, mean (SD)	1.00 (1.07)	1.00 (1.09)	0.001	1.00 (1.04)	1.00 (1.06)	0.004
No. of hospitalizations, mean (SD)	0.20 (0.56)	0.20 (0.55)	0.005	0.20 (0.54)	0.20 (0.52)	0.005
Length of stay <30 d before cohort entry, mean (SD)	0.10 (1.01)	0.10 (0.94)	0.005	0.10 (1.01)	0.10 (0.86)	0.003
No. of emergency visits, mean (SD)	0.70 (1.76)	0.70 (1.81)	0	0.70 (1.73)	0.70 (1.67)	0.010
No. of distinct medications, mean (SD)	13.80 (5.97)	13.80 (5.68)	0.001	13.10 (5.69)	13.00 (5.68)	0.007

Abbreviations: ACEI, angiotensin-converting enzyme inhibitor; ARB, angiotensin receptor blocker; ASMD, absolute standardized mean difference; CKD, chronic kidney disease; COPD, chronic obstructive pulmonary disease; GLP-1RA, glucagon-like peptide-1 receptor agonist; HbA_1c_, hemoglobin A_1c_; MI, myocardial infarction; PCSK9, proprotein convertase subtilisin-kexin type 9 serine protease.

^a^ Baseline characteristics were measured on the index date (cohort entry date) and during the 12 months before the index date, unless otherwise stated.

^b^ For ASMD, values <0.100 were considered to have satisfactory balance.^24^

^c^ Includes American Indian or Alaska Native and unknown race and/or ethnicity.

^d^ Overweight was defined as body mass index (BMI; calculated as weight in kilograms divided by height in meters squared) of 25.0 to less than 30.0; obesity was defined as BMI of 30.0 or higher, history of bariatric surgery, or medications approved for weight loss.

^e^ Concurrent use on the index date was defined as the overlap of days’ supply of baseline medication with the cohort entry date.

### Primary Outcomes

After PS matching, the approximate median treatment duration was 5 months (IQR, 3-10 months) in both cohort 1 and cohort 2. The most common reasons for the end of follow-up were treatment discontinuation (22 344 patients [48.8%]) in cohort 1 and the end of the study period (20 120 patients [44.1%]) in cohort 2 (eTable 2 in the Supplement).

Among those in the empagliflozin vs liraglutide cohort, after PS matching, we identified 29.0 modified MACEs/1000 person-years (PYs) in those initiating empagliflozin and 31.9 modified MACEs/1000 PYs in those initiating liraglutide, which corresponded to an HR of 0.90 (95% CI, 0.79-1.03) and an RD of −2.92/1000 PYs (95% CI, −6.87 to 1.01/1000 PYs) (Table 2). Patients who initiated empagliflozin also had a lower incidence rate of HHF vs those who initiated liraglutide (8.4 events/1000 PYs vs 12.6 events/1000 PYs), with an HR of 0.66 (95% CI, 0.52-0.82) and an RD of −4.16/1000 PYs (95% CI, −6.50 to −1.86/1000 PYs).

**Table 2.** Incidence Rates and Treatment Effect Estimates for Propensity Score–Matched Participants Initiating Treatment With Empagliflozin vs Liraglutide or Sitagliptin

Outcome	Total No. of events (IR/1000 PYs)		Empagliflozin vs comparator	
	Empagliflozin	Comparator	HR (95% CI)	RD/1000 PYs (95% CI)
**Empagliflozin vs liraglutide (n = 22 894 matched pairs)**				
Primary				
Modified MACE^a^	453 (29.0)	466 (31.9)	0.90 (0.79 to 1.03)	−2.92 (−6.87 to 1.01)
HHF (specific)	132 (8.4)	184 (12.6)	0.66 (0.52 to 0.82)	−4.16 (−6.50 to −1.86)
Secondary				
HHF (broad)	633 (40.9)	721 (50.0)	0.81 (0.73 to 0.91)	−9.16 (−14.02 to −4.33)
All-cause mortality	208 (13.2)	198 (13.5)	0.97 (0.79 to 1.17)	−0.25 (−2.85 to 2.35)
MI	158 (10.1)	188 (12.8)	0.78 (0.63 to 0.97)	−2.76 (−5.20 to −0.36)
Stroke	132 (8.4)	114 (7.8)	1.08 (0.84 to 1.39)	0.64 (−1.39 to 2.67)
**Empagliflozin vs sitagliptin (n = 22 812 matched pairs)**				
Primary				
Modified MACE^a^	382 (26.7)	583 (39.1)	0.68 (0.60 to 0.77)	−12.36 (−16.53 to −8.22)
HHF (specific)	112 (7.8)	259 (17.3)	0.45 (0.36 to 0.56)	−9.49 (−12.08 to −6.96)
Secondary				
HHF (broad)	556 (39.2)	881 (60.0)	0.64 (0.58 to 0.72)	−20.82 (−25.97 to −15.70)
All-cause mortality	162 (11.3)	263 (17.5)	0.64 (0.53 to 0.78)	−6.19 (−8.94 to −3.47)
MI	147 (10.3)	218 (14.6)	0.70 (0.57 to 0.87)	−4.31 (−6.87 to −1.77)
Stroke	111 (7.7)	136 (9.1)	0.85 (0.66 to 1.09)	−1.32 (−3.43 to 0.78)

Abbreviations: HHF, hospitalization for heart failure; HR, hazard ratio; IR, incidence rate; MACE, major adverse cardiovascular event; MI, myocardial infarction; PY, person-year; RD, rate difference.

^a^ Includes hospitalization for MI, ischemic stroke, or hemorrhagic stroke and all-cause mortality.

**Figure 1.**
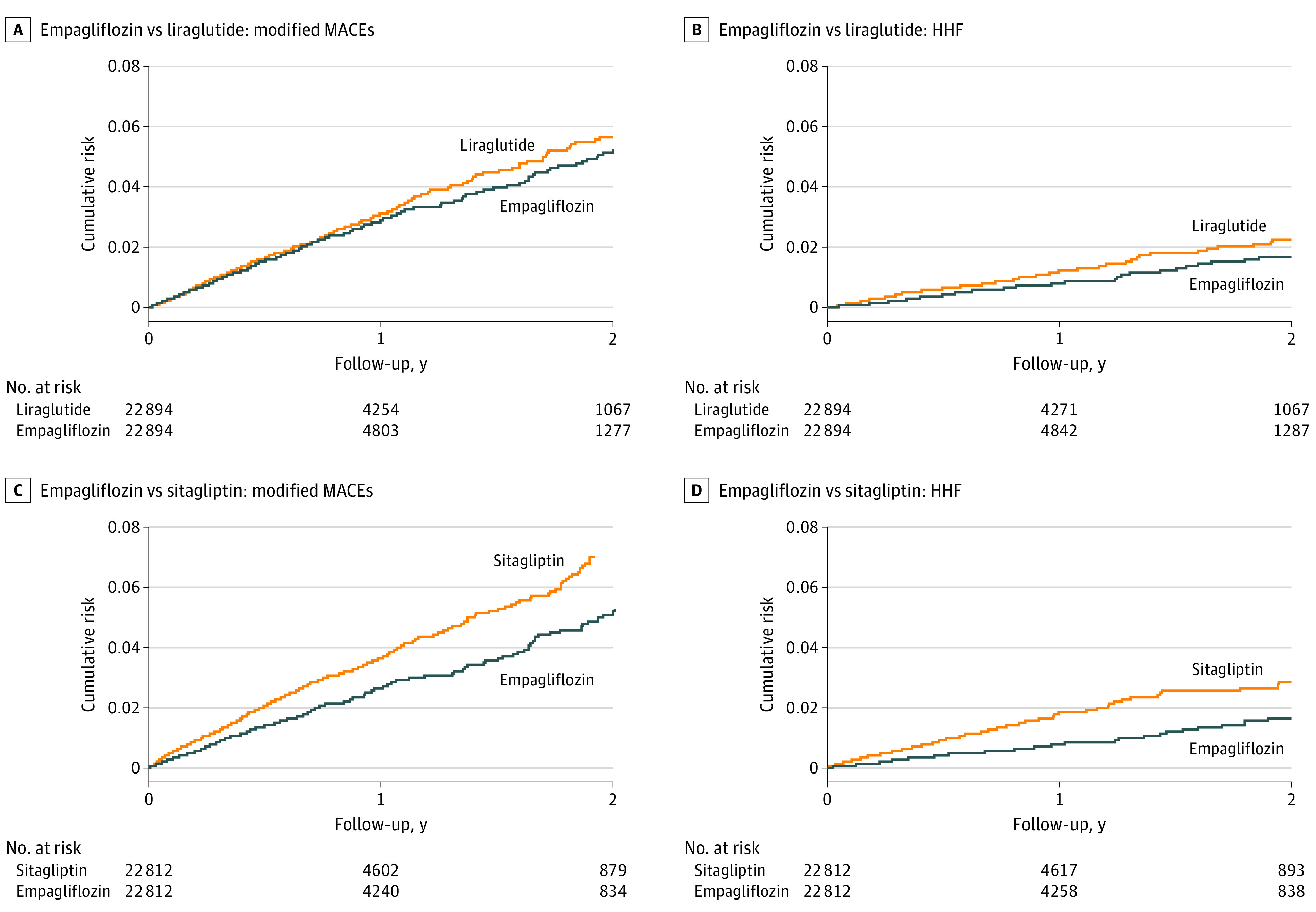
Cumulative Incidence of Primary Outcomes Follow-up time was from drug initiation until discontinuation or drug switching, gap in Medicare enrollment (>30 days), or death. Modified major adverse cardiovascular events (MACEs) include hospitalization for myocardial infarction, ischemic stroke, or hemorrhagic stroke and all-cause death. HHF indicates hospitalization for heart failure.

Relative to sitagliptin, after PS matching, empagliflozin was associated with reductions in the incidence rate of both modified MACEs (26.7/1000 PYs vs 39.1/1000 PYs; HR, 0.68 [95% CI, 0.60-0.77]; RD, −12.36/1000 PYs [95% CI, −16.53 to −8.22/1000 PYs]) and HHF (7.8 events/1000 PYs vs 17.3 events/1000 PYs; HR, 0.45 [95% CI, 0.36-0.56]; RD, −9.49/1000 PYs [95% CI, −12.08 to −6.96/1000 PYs]). The cumulative risks of outcome events as a function of follow-up time for both comparisons are shown in Figure 1.

### Subgroup Analyses

**Figure 2.**
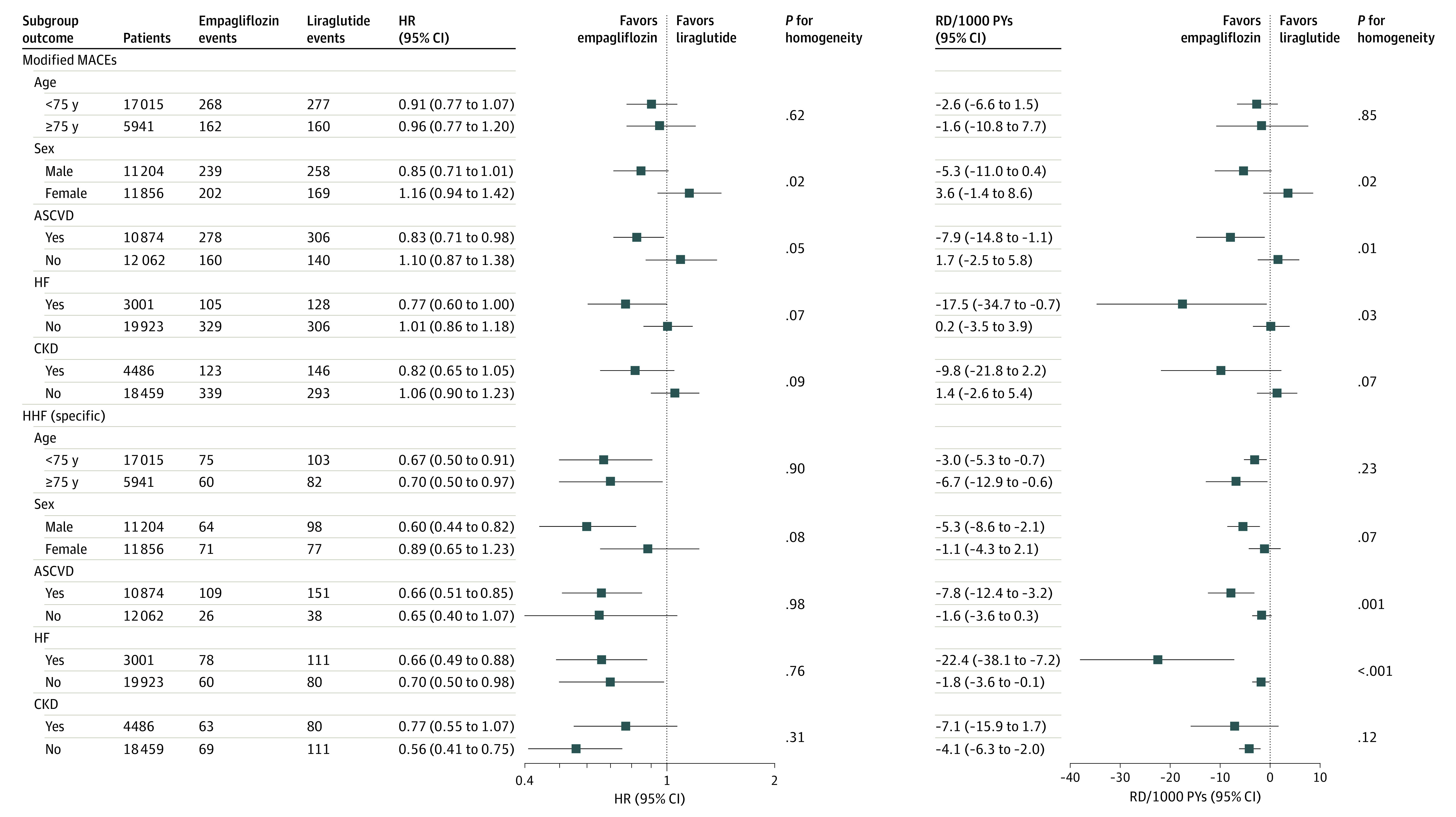
Subgroup Analyses of Propensity Score–Matched Patients Initiating Treatment With Empagliflozin vs Liraglutide Modified major adverse cardiovascular events (MACEs) include hospitalization for myocardial infarction, ischemic stroke, or hemorrhagic stroke and all-cause mortality. ASCVD indicates atherosclerotic cardiovascular disease; CKD, chronic kidney disease; HF, heart failure; HHF, hospitalization for heart failure; HR, hazard ratio; PY, person-year; and RD, rate difference.

For the empagliflozin vs liraglutide cohort, results stratified by age (<75 years vs ≥75 years) were similar to the overall findings (modified MACE: HR, 0.91 [95% CI, 0.77-1.07] vs 0.96 [95% CI, 0.77-1.20]; RD, −2.55/1000 PYs [95% CI, −6.57 to 1.47/1000 PYs] vs −1.59/1000 PYs [95% CI, −10.82 to 7.65/1000 PYs]; HHF: HR, 0.67 [95% CI, 0.50-0.91] vs 0.70 [95% CI, 0.50-0.97]; RD, −3.02/1000 PYs [95% CI, −5.32 to −0.73/1000 PYs] vs −6.74/1000 PYs [95% CI, −12.89 to −0.58/1000 PYs]) (Figure 2). When stratified by sex, we observed larger RDs (absolute risk reductions) with respect to the modified MACE outcome in male vs female patients, with *P* = .02 for homogeneity (RD, −5.29/1000 PYs [95% CI, −11.00 to 0.35/1000 PYs] vs 3.59/1000 PYs [95% CI, −1.41 to 8.61/1000 PYs]). The HR and RD estimates were more similar for HHF outcomes between male and female patients (HR, 0.60 [95% CI, 0.44-0.82] vs 0.89 [95% CI, 0.65-1.23]; *P* = .08 for homogeneity; RD, −5.32/1000 PYs [95% CI, −8.63 to −2.11/1000 PYs] vs −1.11/1000 PYs [95% CI, −4.30 to 2.05/1000 PYs]; *P* = .07 for homogeneity). For cardiorenal subgroups, empagliflozin was associated with a lower risk of the modified MACE outcome relative to liraglutide in patients with baseline ASCVD (HR, 0.83 [95% CI, 0.71-0.98]; RD, −7.91/1000 PYs [95% CI, −14.84 to −1.05/1000 PYs]) or baseline HF (HR, 0.77 [95% CI, 0.60-1.00]; RD, −17.53/1000 PYs [95% CI, −34.66 to −0.69/1000 PYs]), and estimates were closer to the null in those with CKD (HR, 0.82 [95% CI, 0.65-1.05]; RD, −9.78/1000 PYs [95% CI, −21.84 to 2.17/1000 PYs]), with varying degrees of precision of the estimates. Estimates revealed no benefits for empagliflozin with respect to the modified MACE outcome in those without baseline ASCVD (HR, 1.10 [95% CI, 0.87-1.38]; RD, 1.66/1000 PYs [95% CI, −2.45 to 5.78/1000 PYs]), baseline HF (HR, 1.01 [95% CI, 0.86-1.18]; RD, 0.20/1000 PYs [95% CI, −3.49 to 3.88/1000 PYs]), or CKD (HR, 1.06 [95% CI, 0.90-1.23]; RD, 1.41/1000 PYs [95% CI, −2.60 to 5.40/1000 PYs]).

Patients initiating empagliflozin vs liraglutide had a lower risk of HHF, regardless of ASCVD, HF, or CKD history, although absolute risk reductions were larger, with *P* = .001 for homogeneity in those with vs without baseline ASCVD (RD, −7.77/1000 PYs [95% CI, −12.44 to −3.21/1000 PYs] vs −1.62/1000 PYs [95% CI, −3.58 to 0.27/1000 PYs]) and *P* < .001 for homogeneity in those with vs without baseline HF (RD, −22.43/1000 PYs [95% CI, −38.07 to −7.18/1000 PYs] vs −1.82/1000 PYs [95% CI, −3.58 to −0.11/1000 PYs]). The RDs for HHF were more similar in patients with vs without baseline CKD than in other subgroups (RD, −7.05/1000 PYs [95% CI, −15.91 to 1.66/1000 PYs] vs −4.08/1000 PYs [95% CI, −6.28 to −1.95/1000 PYs]).

**Figure 3.**
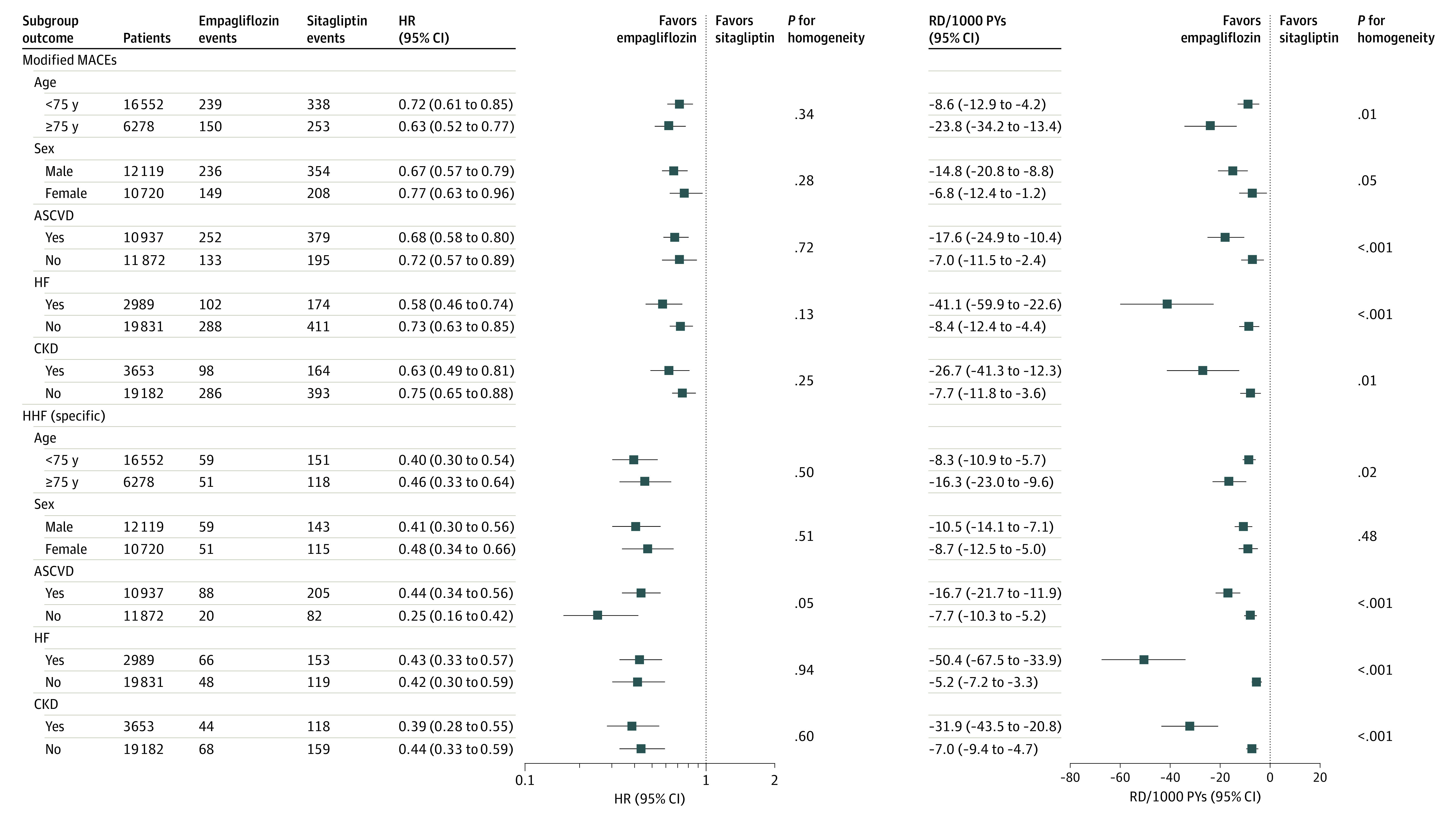
Subgroup Analyses of Propensity Score–Matched Patients Initiating Treatment With Empagliflozin vs Sitagliptin Modified major adverse cardiovascular events (MACEs) include hospitalization for myocardial infarction, ischemic stroke, or hemorrhagic stroke and all-cause mortality. ASCVD indicates atherosclerotic cardiovascular disease; CKD, chronic kidney disease; HF, heart failure; HHF, hospitalization for heart failure; HR, hazard ratio; PY, person-year; and RD, rate difference.

Compared with sitagliptin, empagliflozin was associated with reductions in the risk of the modified MACE outcome and HHF, regardless of age, sex, or history of ASCVD, HF, or CKD (Figure 3). However, the absolute benefits associated with empagliflozin were larger in adults 75 years or older vs adults younger than 75 years (modified MACE: RD, −23.80/1000 PYs [95% CI, −34.24 to −13.36/1000 PYs] vs −8.58/1000 PYs [95% CI, −12.93 to −4.24/1000 PYs]; HHF: RD, −16.26/1000 PYs [95% CI, −22.96 to −9.56/1000 PYs vs −8.29/1000 PYs [95% CI, −10.89 to −5.68/1000 PYs]), patients with vs without baseline ASCVD (modified MACE: RD, −17.63/1000 PYs [95% CI, −24.92 to −10.40/1000 PYs] vs −6.95/1000 PYs [95% CI, −11.54 to −2.41/1000 PYs]; HHF: RD, −16.72/1000 PYs [95% CI, −21.71 to −11.86/1000 PYs] vs −7.66/1000 PYs [95% CI, −10.27 to −5.22/1000 PYs]), patients with vs without baseline HF (modified MACE: RD, −41.06/1000 PYs [95% CI, −59.88 to −22.61/1000 PYs] vs −8.38/1000 PYs [95% CI, −12.42 to −4.35/1000 PYs]; HHF: RD, −50.38/1000 PYs [95% CI, −67.46 to −33.89/1000 PYs] vs −5.22/1000 PYs [95% CI, −7.22 to −3.30/1000 PYs]), and patients with vs without CKD (modified MACE: RD, −26.70/1000 PYs [95% CI, −41.26 to −12.33/1000 PYs] vs −7.71/1000 PYs [95% CI, −11.84 to −3.60/1000 PYs]; HHF: RD, −31.94/1000 PYs [95% CI, −43.52 to −20.81/1000 PYs] vs −7.02/1000 PYs [95% CI, −9.42 to −4.68/1000 PYs]).

### Secondary Outcomes

Estimates for the HHF outcome based on any discharge position (HHF broad) were similar to those based on the primary discharge position for both empagliflozin vs liraglutide (40.9 events/1000 PYs vs 50.0 events/1000 PYs; HR, 0.81 [95% CI, 0.73-0.91]; RD, −9.16/1000 PYs [95% CI, −14.02 to –4.33/1000 PYs]) and empagliflozin vs sitagliptin (39.2 events/1000 PYs vs 60.0 events/1000 PYs; HR, 0.64 [95% CI, 0.58-0.72]; RD, −20.82/1000 PYs [95% CI, −25.97 to −15.70/1000 PYs]) (Table 2). When compared with patients who initiated liraglutide, those who initiated empagliflozin had a lower risk of MI (HR, 0.78 [95% CI, 0.63-0.97]; RD, −2.76/1000 PYs [95% CI, −5.20 to −0.36/1000 PYs]). Relative to sitagliptin, empagliflozin was associated with a lower risk of death (HR, 0.64 [95% CI, 0.53-0.78]; RD, −6.19/1000 PYs [95% CI, −8.94 to −3.47/1000 PYs]) and MI (HR, 0.70 [95% CI, 0.57-0.87]; RD, −4.31/1000 PYs [95% CI, −6.87 to −1.77/1000 PYs]).

### Sensitivity Analyses

Analyses based on intention-to-treat follow-up (up to 2 years), a grace period of 30 days, and other sensitivity analyses revealed estimates similar to the primary findings (eTable 3 in the Supplement). When we compared empagliflozin with the class of GLP-1RAs or DPP-4 inhibitors, results were consistent with main findings, both overall and across subgroups (eTable 4, eFigure 3, and eFigure 4 in the Supplement). However, estimates for the modified MACE outcome for empagliflozin vs GLP-1RAs were closer to the null in patients with ASCVD (HR, 0.92 [95% CI, 0.80-1.06]; RD, −3.63/1000 PYs [95% CI, −9.59 to 2.34/1000 PYs]), HF (HR, 0.89 [95% CI, 0.71-1.12]; RD, −7.97/1000 PYs [95% CI, −22.72 to 6.79/1000 PYs]) when compared with the findings in the empagliflozin vs liraglutide comparison.

Results from bias analyses revealed that the scenario of an unadjusted continuous confounder (eg, hemoglobin A_1c_) with a strong effect on the outcome (relative risk for primary cardiovascular outcomes of 1.3 for every 1% increase in hemoglobin A_1c_)^30^ would produce estimates fairly consistent with the primary findings (eFigure 5 in the Supplement).

## Discussion

In this large comparative effectiveness study, empagliflozin was associated with a lower risk of HHF relative to liraglutide and a lower risk of both the modified MACE outcome and HHF relative to sitagliptin. Compared with liraglutide, we observed a benefit of empagliflozin with respect to the risk of the modified MACE outcome in patients with a history of ASCVD or HF. The risk of HHF was consistently lower in those who initiated empagliflozin vs liraglutide across all subgroups, with larger absolute benefits in patients with ASCVD and HF. Compared with sitagliptin, the absolute benefit with empagliflozin for both the modified MACE outcome and HHF were also larger in patients with ASCVD, HF, and CKD compared with patients without those conditions and in adults 75 years and older compared with adults younger than 75 years.

To our knowledge, this study was one of the few to date to compare the cardiovascular effectiveness of empagliflozin vs alternative individual agents. Although both SGLT-2 inhibitors and GLP-1RAs have demonstrated cardiovascular benefits,^6,12^ such benefits were not consistently seen across individual SGLT-2 inhibitors or GLP-1RAs in clinical trials (eg, dapagliflozin and ertugliflozin [SGLT-2 inhibitors] and exenatide and lixisenatide [GLP-1RAs] did not have MACE benefit).^5,31,32^ Although these findings could be explained by the differences in study design and population characteristics (eg, varying prevalence of ASCVD or treatment discontinuation rates),^5,31,32^ effect heterogeneity across individual agents is a potential alternative explanation. With the increasing use of empagliflozin in recent years,^11^ evidence on its effectiveness in routine care has become relevant to guide treatment decision-making.

Consistent with a meta-analysis of placebo-controlled clinical trials,^4^ we found that benefits for the modified MACE outcome associated with empagliflozin relative to liraglutide were mostly observed in patients with baseline ASCVD and HF. Although the meta-analysis of placebo-controlled clinical trials suggested that benefit for MACEs with SGLT-2 inhibitors or GLP-1RAs did not differ by history of HF,^4^ we observed larger benefit for the modified MACE outcome with empagliflozin relative to liraglutide in patients with HF history than in those without it. Findings from a Danish cohort study^9^ were also similar to ours, although the study populations, outcome definitions, and choice of baseline CVD subgroup definition were different.

In contrast to findings from the post hoc analyses of the EMPA-REG OUTCOME (Empagliflozin Cardiovascular Outcome Event Trial in Type 2 Diabetes Mellitus Patients) and LEADER (Liraglutide Effect and Action in Diabetes: Evaluation of Cardiovascular Outcome Results) clinical trials, we observed no variation in the risk of the modified MACE outcome by age among patients initiating empagliflozin vs liraglutide.^4,7^ We also observed potential heterogeneity in estimates for the modified MACE outcome for empagliflozin vs liraglutide by male and female patients. Because most participants in cardiovascular outcome trials are male, future studies are warranted to explore whether empagliflozin benefits are similar in female patients. With regard to subgroups by CKD status, we observed larger absolute RDs in favor of empagliflozin in patients with a history of CKD with respect to both modified MACE and HHF outcomes, a finding also suggested by a previous meta-analysis of clinical trials.^4^

Our findings on empagliflozin relative to sitagliptin were consistent with findings from clinical trials, likely because DPP-4 inhibitors, especially sitagliptin, are largely neutral with respect to cardiovascular outcomes.^13^ Relative to sitagliptin, we observed larger absolute benefit of empagliflozin for both modified MACE and HHF outcomes in subgroups of patients with ASCVD, HF, and CKD and patients 75 years and older.

Several methodological aspects need to be considered when interpreting the findings of this study. A new-user active-comparator cohort study design^33^ reduces the potential for time-related biases and the confounding associated with long-term medication use (ie, prevalent use).^34^ A previous new-user active-comparator study^35^ controlling for more than 100 claims-based participant baseline characteristics was able to balance biomarkers and laboratory results obtained from the electronic medical records in patients with T2D. Previous studies aiming to emulate the results of randomized clinical trials reported that study investigators were able to replicate the findings of the cardiovascular outcome clinical trials (and estimate the findings of the ongoing clinical trial before they were finished) using study designs and analytical strategies similar to ours.^36,37^ Nevertheless, residual confounding cannot be entirely ruled out.

### Limitations

This study has several limitations. Our outcome definitions were based on Medicare claims-based data. However, these algorithms have been previously validated, and outcome definitions with high specificity have been found to produce valid relative measures of association.^14,15,16,17,18^ We also could not evaluate cardiovascular-specific mortality; however, almost 70% of causes of death in older Medicare populations are due to cardiovascular causes,^38^ which could be even higher in the populations included in our study because patients with a history of cancer at baseline were excluded.

Another limitation of our study was the brief median treatment duration in the cohorts, which was approximately 5 to 6 months and reflected medication use patterns in routine clinical practice. However, analyses without censoring for treatment changes and with up to 2 years of follow-up data produced findings similar to those of the primary analyses. Due to the lack of randomization, the possibility of residual confounding cannot be entirely eliminated.

## Conclusions

In this comparative effectiveness cohort study of older adults, relative to liraglutide, empagliflozin was associated with a lower risk of the modified MACE outcome in patients with a history of ASCVD and HF and potential heterogeneity of estimates in male vs female patients, whereas reductions in the risk of HHF were observed across most major patient subgroups. Compared with sitagliptin, empagliflozin was associated with a lower risk of both modified MACE and HHF outcomes across all subgroups. The absolute benefits of empagliflozin were generally larger in patients with a history of ASCVD, HF, and CKD. These findings suggest that older adults with T2D might benefit more from empagliflozin vs liraglutide or sitagliptin with respect to the risk of HHF; with respect to the risk of major cardiovascular events, empagliflozin might be preferable to liraglutide only in patients with the history of CVD and to sitagliptin across all patient subgroups.

## Notes

Supplement. **eFigure 1.** Flow Diagram for Empagliflozin vs Liraglutide Cohort **eFigure 2.** Flow Diagram for Empagliflozin vs Sitagliptin Cohort **eFigure 3.** Hazard Ratios and Rate Differences for 1:1 PS-Matched Initiators of Empagliflozin vs GLP-1RA Across Patient Subgroups **eFigure 4.** Hazard Ratios and Rate Differences for 1:1 PS-Matched Initiators of Empagliflozin vs DPP-4 Inhibitor Across Patient Subgroups **eFigure 5.** Bias Analyses **eTable 1.** Unmatched Distribution of Baseline Characteristics **eTable 2.** Follow-up Time and Censoring Reason for Primary Outcomes Between 1:1 PS-Matched Initiators of Empagliflozin vs Liraglutide or Sitagliptin **eTable 3.** Sensitivity Analyses for 1:1 PS-Matched Initiators of Empagliflozin vs Liraglutide or Sitagliptin **eTable 4.** Number of Events, Incidence Rates, and Treatment Effect Estimates for 1:1 PS-Matched Initiators of Empagliflozin vs GLP-1RA or DPP-4 Inhibitor in the Overall Population
