# Supplementary material for: Comparative Effectiveness of Empagliflozin vs Liraglutide or Sitagliptin in Older Adults With Diverse Patient Characteristics
Source: JAMA Netw Open. 2022 Oct 20;5(10):e2237606. doi: 10.1001/jamanetworkopen.2022.37606 (PMC9585433; doi:10.1001/jamanetworkopen.2022.37606)
Supplement: Supplement. — eFigure 1. Flow Diagram for Empagliflozin vs Liraglutide Cohort eFigure 2. Flow Diagram for Empagliflozin vs Sitagliptin Cohort eFigure 3. Hazard Ratios and Rate Differences for 1:1 PS-Matched Initiators of Empagliflozin vs GLP-1RA Across Patient Subgroups eFigure 4. Hazard Ratios and Rate Differences for 1:1 PS-Matched Initiators of Empagliflozin vs DPP-4 Inhibitor Across Patient Subgroups eFigure 5. Bias Analyses eTable 1. Unmatched Distribution of Baseline Characteristics eTable 2. Follow-up Time and Censoring Reason for Primary Outcomes Between 1:1 PS-Matched Initiators of Empagliflozin vs Liraglutide or Sitagliptin eTable 3. Sensitivity Analyses for 1:1 PS-Matched Initiators of Empagliflozin vs Liraglutide or Sitagliptin eTable 4. Number of Events, Incidence Rates, and Treatment Effect Estimates for 1:1 PS-Matched Initiators of Empagliflozin vs GLP-1RA or DPP-4 Inhibitor in the Overall Population [file jamanetwopen-e2237606-s001.pdf]

Supplementary Online Content

Htoo PT, Tesfaye H, Schneeweiss S, et al. Comparative effectiveness of empagliflozin vs liraglutide or sitagliptin in older adults with diverse patient characteristics. *JAMA Netw Open*. 2022;5(10):e2237606. doi:10.1001/jamanetworkopen.2022.37606

eFigure 1. Flow Diagram for Empagliflozin vs Liraglutide Cohort

eFigure 2. Flow Diagram for Empagliflozin vs Sitagliptin Cohort

eFigure 3. Hazard Ratios and Rate Differences for 1:1 PS-Matched Initiators of Empagliflozin vs GLP-1RA Across Patient Subgroups

eFigure 4. Hazard Ratios and Rate Differences for 1:1 PS-Matched Initiators of Empagliflozin vs DPP-4 Inhibitor Across Patient Subgroups

eFigure 5. Bias Analyses

eTable 1. Unmatched Distribution of Baseline Characteristics

eTable 2. Follow-up Time and Censoring Reason for Primary Outcomes Between 1:1 PS-Matched Initiators of Empagliflozin vs Liraglutide or Sitagliptin

eTable 3. Sensitivity Analyses for 1:1 PS-Matched Initiators of Empagliflozin vs Liraglutide or Sitagliptin

eTable 4. Number of Events, Incidence Rates, and Treatment Effect Estimates for 1:1 PS-Matched Initiators of Empagliflozin vs GLP-1RA or DPP-4 Inhibitor in the Overall Population

This supplementary material has been provided by the authors to give readers additional information about their work.

© 2022 Htoo PT et al. *JAMA Network Open*.

eFigure 1. Flow Diagram for Empagliflozin vs Liraglutide Cohort

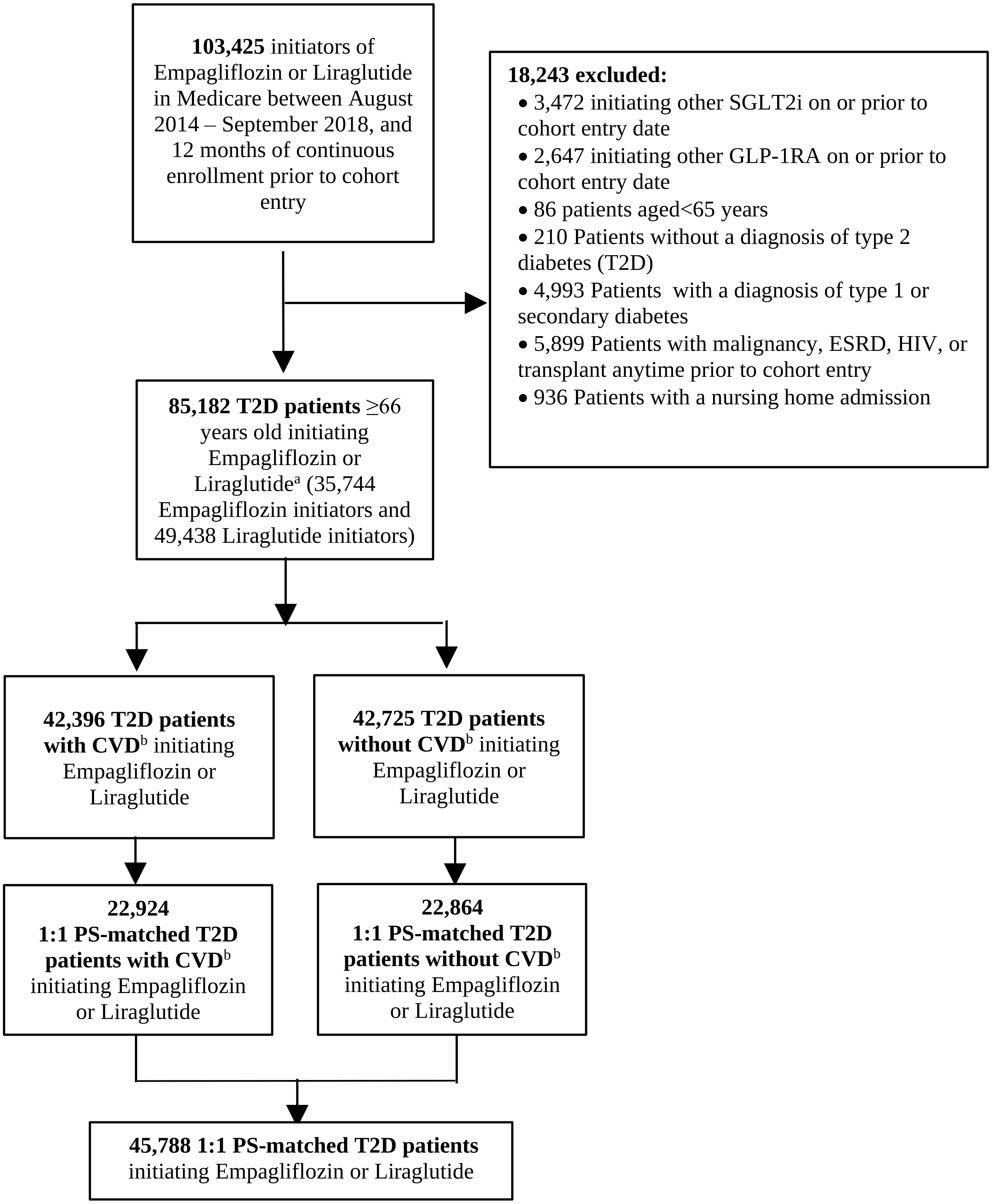

SGLT2i: sodium-glucose cotransporter 2 inhibitors; GLP-1RA: glucagon-like peptide-1 receptor agonists; CVD: cardiovascular disease; PS: propensity score; T2D: type 2 diabetes

[a] 61 patients did not begin follow-up since they were not alive on the beginning of follow-up (one day after cohort entry) [b] Defined as history of myocardial infarction, angina, coronary atherosclerosis and other forms of chronic ischemic heart disease, coronary procedure, heart failure, ischemic stroke, peripheral arterial disease or surgery, lower extremity amputation.

© 2022 Htoo PT et al. *JAMA Network Open*.

eFigure 2. Flow Diagram for Empagliflozin vs Sitagliptin Cohort

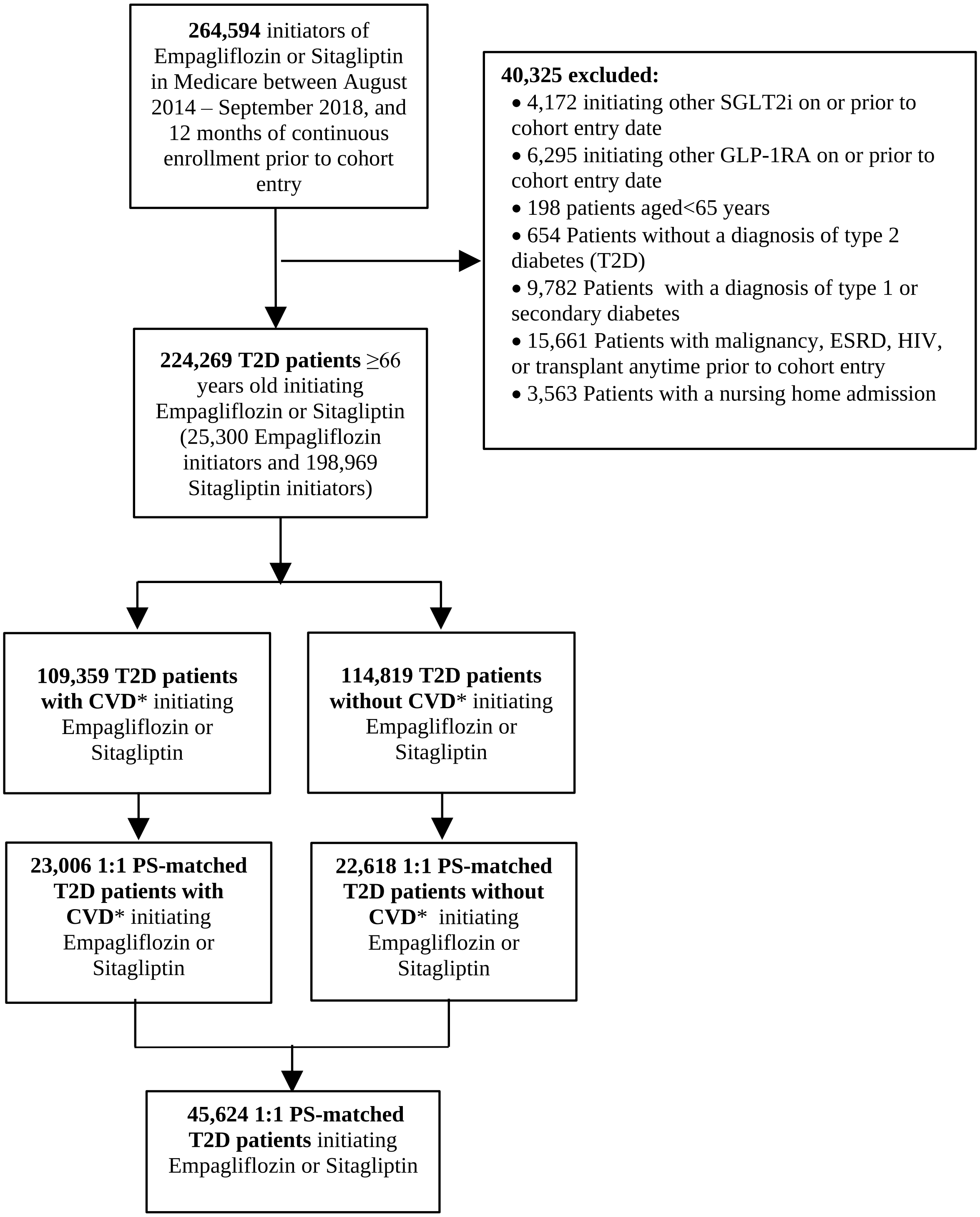

SGLT2i: sodium-glucose cotransporter 2 inhibitors; GLP-1RA: glucagon-like peptide-1 receptor agonists; CVD: cardiovascular disease; PS: propensity score; T2D: type 2 diabetes

[a] 91 patients did not begin follow-up since they were not alive on the beginning of follow-up (one day after cohort entry)

[b] Defined as history of myocardial infarction, angina, coronary atherosclerosis and other forms of chronic ischemic heart disease, coronary procedure, heart failure, ischemic stroke, peripheral arterial disease or surgery, lower extremity amputation

© 2022 Htoo PT et al. *JAMA Network Open*.

eFigure 3. Hazard Ratios and Rate Differences for 1:1 PS-Matched Initiators of Empagliflozin vs GLP-1RA Across Patient Subgroups

Outcomes (subgroups)	Empagliflozin Events (N)	GLP-1RA Events (N)	HR (95% CI)	Empagliflozinvs. GLP-1RA	p-value for homogeneity	RD/1000PY (95% CI)	Empagliflozin vs. GLP-1RA	p-value for homogeneity
**Modified MACE**								
Age<75	338 (22,844)	353 (22,844)	0.90 (0.77, 1.04)		0.95	-2.6 (-6.1, 0.9)		0.98
Age>=75	229 (8,935)	232 (8,935)	0.93 (0.77, 1.11)			-3.0 (-10.1, 4.7)		
Male	323 (16,248)	340 (16,248)	0.87 (0.75, 1.02)		0.96	-4.3 (-9.0, 0.5)		0.93
Female	255 (15,567)	254 (15,567)	0.96 (0.81, 1.15)			-1.0 (-5.7, 3.7)		
ASCVD +	390 (15,062)	394 (15,062)	0.92 (0.80, 1.06)		0.54	-3.6 (-9.6, 2.3)		0.28
ASCVD -	195 (16,626)	186 (16,626)	0.99 (0.81, 1.21)			-0.2 (-3.7, 3.4)		
HF +	151 (4,088)	156 (4,088)	0.89 (0.71, 1.12)		0.52	-8.0 (-22.9, 6.8)		0.33
HF -	430 (27,621)	417 (27,621)	0.97 (0.84, 1.11)			-0.8 (-4.1, 2.4)		
CKD +	143 (5,502)	170 (5,502)	0.82 (0.66, 1.03)		0.19	-9.5 (-20.2, 1.2)		0.11
CKD -	444 (26,276)	421 (26,276)	0.98 (0.86, 1.12)			-0.6 (-4.01, 2.8)		
**HHF (specific)**								
Age<75	82 (22,844)	127 (22,844)	0.60 (0.46, 0.80)		0.24	-3.5 (-5.5, -1.6)		0.88
Age>=75	77 (8,935)	96 (8,935)	0.75 (0.56, 1.01)			-4.4 (-9.2, 0.3)		
Male	77 (16,248)	133 (16,248)	0.53 (0.40, 0.70)		0.10	-6.1 (-8.8, -3.4)		0.10
Female	81 (15,567)	104 (15,567)	0.75 (0.56, 1.00)			-2.8 (-5.7, 0.0)		
ASCVD +	128 (15,062)	186 (15,062)	0.64 (0.51, 0.80)		0.59	-7.6 (-11.5, -3.9)		0.00
ASCVD -	34 (16,626)	44 (16,626)	0.73 (0.46, 1.14)			-1.1 (-2.8, 0.5)		
HF +	96 (4,088)	131 (4,088)	0.67 (0.52, 0.88)		0.90	-19.6 (-32.6, -6.8)		<0.00
HF -	66 (27,621)	90 (27,621)	0.68 (0.50, 0.94)			-1.6 (-3.0, -0.3)		
CKD +	72 (5,502)	106 (5,502)	0.66 (0.49, 0.89)		0.94	-11.2 (-19.4, -3.2)		0.03
CKD -	97 (26,276)	138 (26,276)	0.65 (0.50, 0.84)			-3.0 (-4.8, -1.2)		

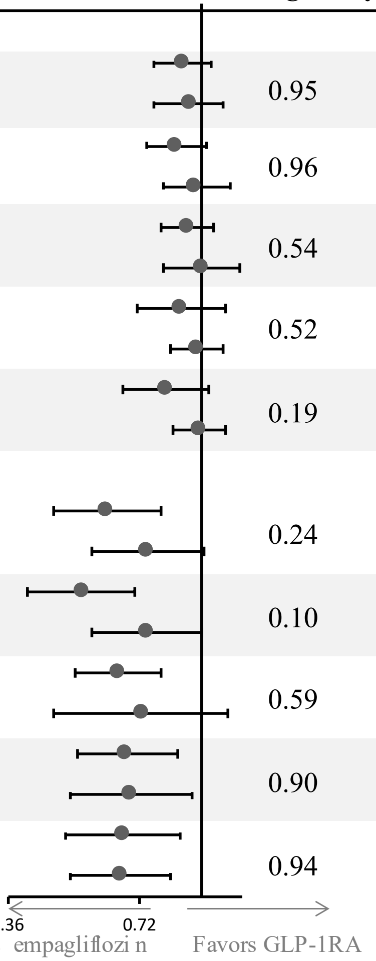

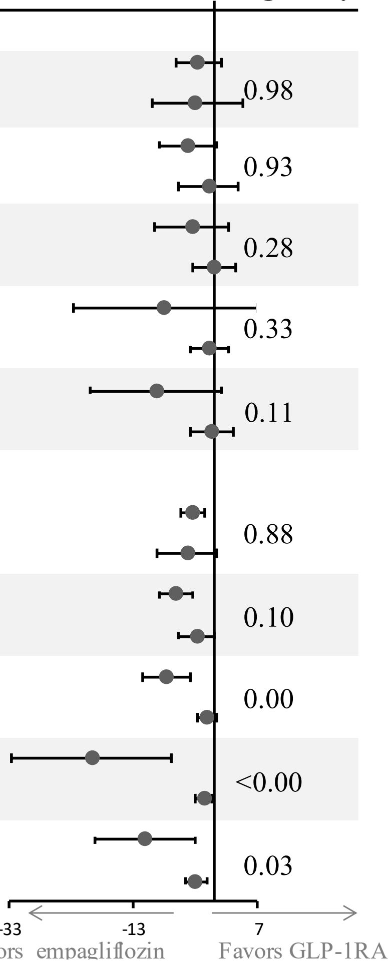

© 2022 Htoo PT et al. *JAMA Network Open*.

eFigure 4. Hazard Ratios and Rate Differences for 1:1 PS-Matched Initiators of Empagliflozin vs DPP-4 Inhibitor Across Patient Subgroups

Outcomes (Subgroups)	Empagliflozin Events (N)	DPP-4i Events (N)	HR (95% CI)	Empagliflozinvs. DPP-4i	p-value for homogeneity	RD/1000PY (95% CI)	Empagliflozinvs. DPP-4i	p-value for homogeneity
**Modified MACE**								
Age<75	238 (16,992)	339 (16,992)	0.71 (0.60, 0.84)		0.52	-8.8 (-13.1, -4.6)		0.24
Age>=75	148 (6,279)	201 (6,279)	0.77 (0.63, 0.96)			-11.9 (-21.7, -2.1)		
Male	237 (12,474)	358 (12,474)	0.66 (0.56, 0.78)		0.71	-15.0 (-21.0, -9.2)		0.26
Female	150 (10,853)	233 (10,853)	0.69 (0.56, 0.85)			-10.4 (-16.1, -4.7)		
ASCVD +	260 (11,273)	377 (11,273)	0.69 (0.59, 0.81)		0.77	-16.6 (-23.7, -9.5)		0.04
ASCVD -	130 (12,041)	200 (12,041)	0.67 (0.53, 0.83)			-8.4 (-13.0, -3.9)		
HF +	100 (3,099)	153 (3,099)	0.67 (0.52, 0.86)		0.84	-28.5 (-45.8, -11.5)		0.04
HF -	291 (20,213)	442 (20,213)	0.68 (0.59, 0.79)			-10.7 (-14.8, -6.7)		
CKD +	101 (3,757)	125 (3,757)	0.84 (0.64, 1.09)		0.25	-8.8 (-22.1, 4.5)		0.89
CKD -	290 (19,519)	419 (19,519)	0.70 (0.61, 0.82)			-9.8 (-14.0, -5.7)		
**HHF (specific)**								
Age<75	64 (16,992)	170 (16,992)	0.38 (0.29, 0.51)		0.00	-9.5 (-12.2, -6.8)		0.23
Age>=75	53 (6,279)	92 (6,279)	0.61 (0.43, 0.85)			-9.5 (-15.7, -3.2)		
Male	62 (12,474)	157 (12,474)	0.39 (0.29, 0.53)		0.19	-11.7 (-15.3, -8.2)		0.08
Female	52 (10,853)	106 (10,853)	0.53 (0.38, 0.73)			-7.2 (-11.0, -3.6)		
ASCVD +	97 (11,273)	218 (11,273)	0.45 (0.35, 0.57)		0.87	-17.3 (-22.4, -12.4)		0.00
ASCVD -	20 (12,041)	48 (12,041)	0.43 (0.25, 0.72)			-3.5 (-5.6, -1.5)		
HF +	69 (3,099)	136 (3,099)	0.51 (0.38, 0.67)		0.79	-37.3 (-53.2, -21.9)		0.00
HF -	46 (20,213)	100 (20,213)	0.48 (0.34, 0.68)			-3.9 (-5.8, -2.2)		
CKD +	46 (3,757)	94 (3,757)	0.50 (0.35, 0.72)		0.82	-20.6 (-31.3, -10.3)		<0.00
CKD -	68 (19,519)	144 (19,519)	0.48 (0.36, 0.64)			-5.9 (-8.2, -3.7)		

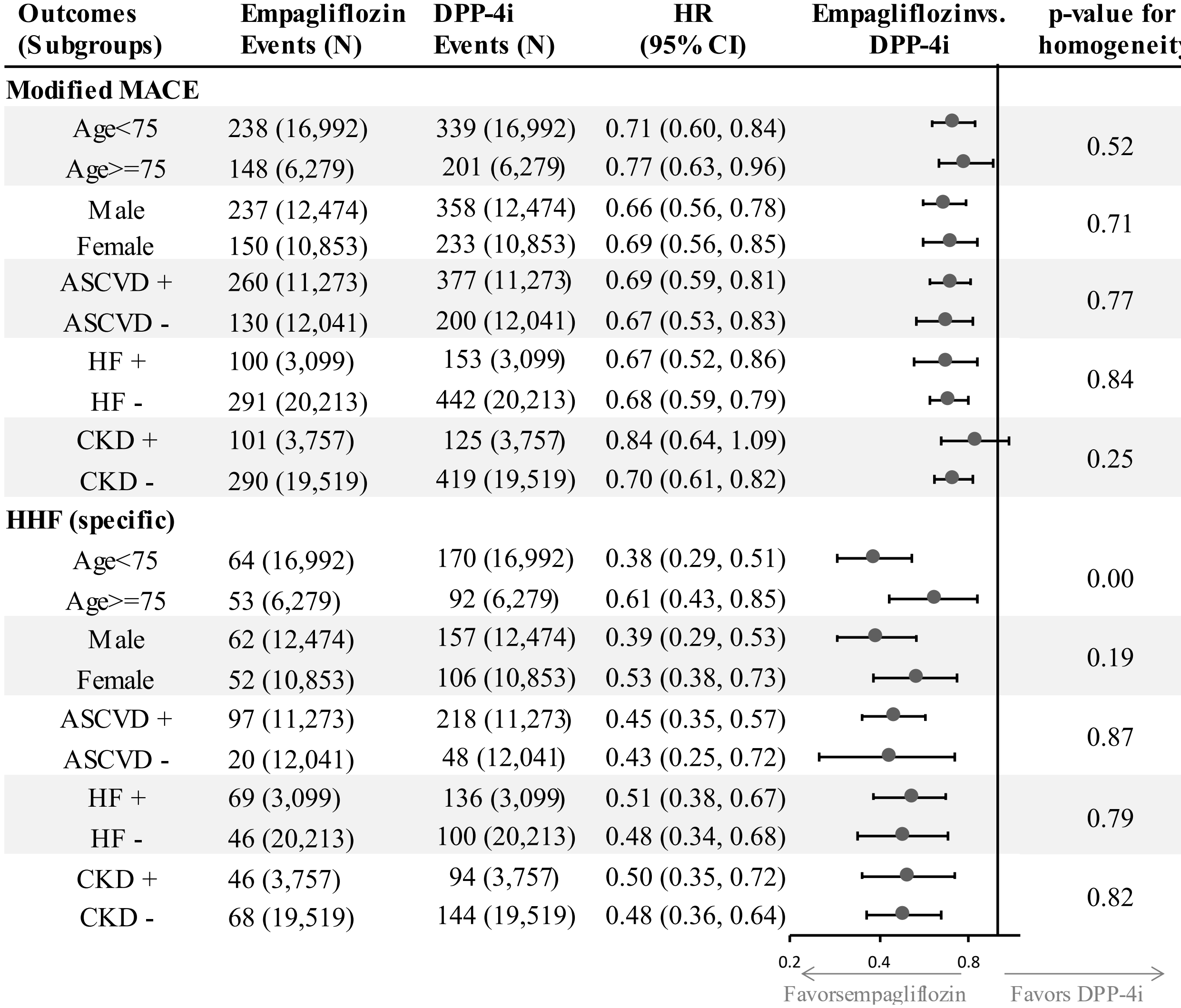

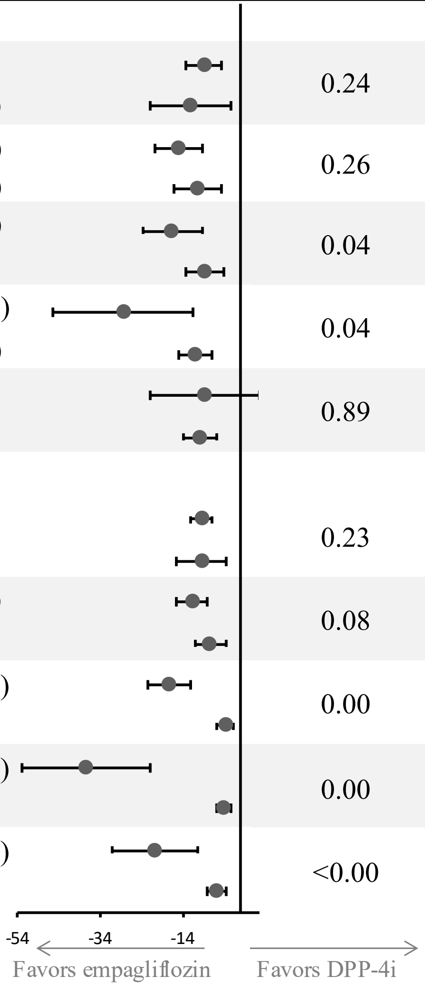

© 2022 Htoo PT et al. *JAMA Network Open*.

eFigure 5. Bias Analyses

Modified MACE outcome[a]	Hospitalization for heart failure
**Empagliflozin vs. Liraglutide**	
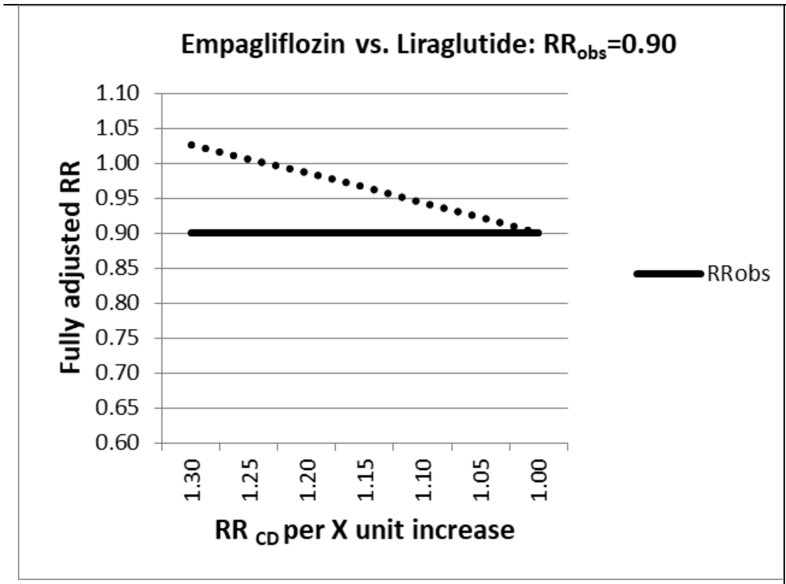	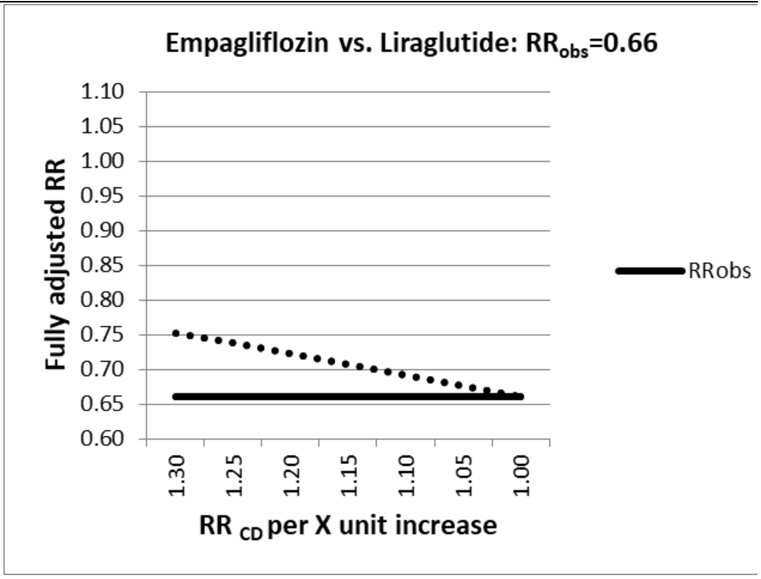
**Empagliflozin vs. Sitagliptin**	
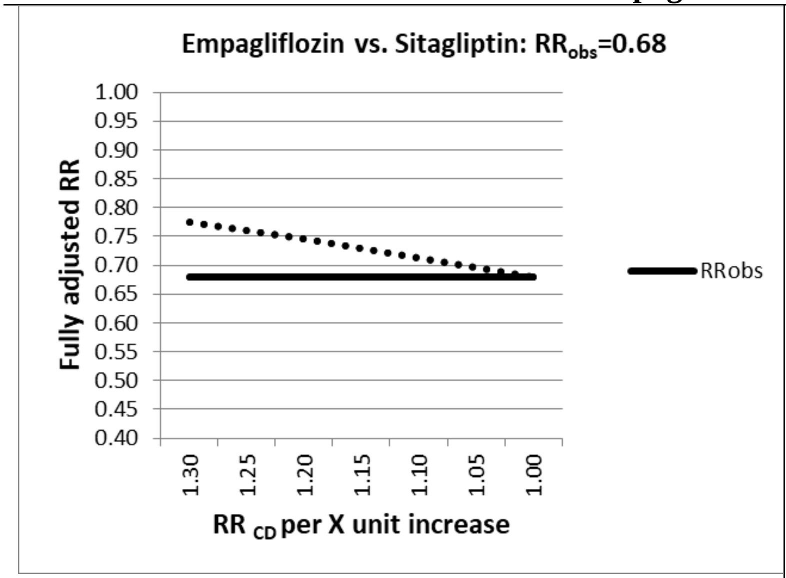	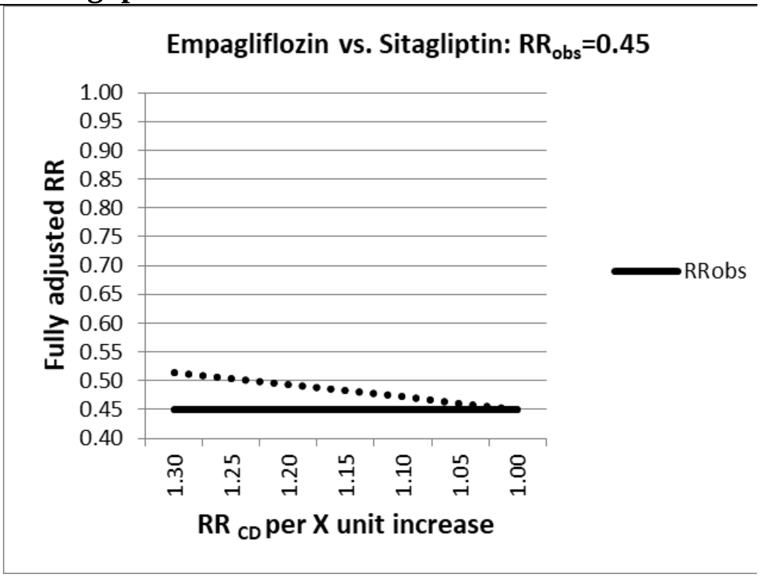

HbA1c: Hemoglobin A1c; MACE: Major Adverse Cardiovascular Events; RR_{obs} : Observed relative risk; RR_{CD} : Relative risk between confounder (HbA1c) and primary cardiovascular outcomes

[a] Hospitalization for myocardial infarction, or ischemic or hemorrhagic stroke; all-cause mortality

Using the observed residual difference in HbA1c between initiators of empagliflozin vs. liraglutide and the observed exposure relative risk (RR_{obs}) = 0.90 and 0.66 for the composite cardiovascular outcome, and HHF respectively (0.68 and 0.45 respectively for sitagliptin comparison), the graphs plot the changes in "true" or fully adjusted relative risk (RR) for a range of associations between the observed HbA1c and the outcome (RR_{CD}). RR_{CD} values were obtained from literature (Gerstein HC, Swedberg K, Carlsson J, et al. The Hemoglobin A1c Level as a Progressive Risk Factor for Cardiovascular Death, Hospitalization for Heart Failure, or Death in Patients With Chronic Heart Failure: An Analysis of the Candesartan in Heart Failure: Assessment of Reduction in Mortality and Morbidity (CHARM) Program. Arch Intern Med. 2008;168(15):1699-1704) and mean HbA1c among empagliflozin vs. comparators were also obtained from prior studies on trial-enrolled participants (Gurgle HE, White K, McAdam-Marx C. SGLT2 inhibitors or GLP-1 receptor agonists as second-line therapy in type 2 diabetes: patient selection and perspectives. Vasc Health Risk Manag. 2016;12:239-249. Published 2016 Jun 4. doi:10.2147/VHRM.S83088). Overall, fully adjusted effect estimates were fairly robust even under extreme scenarios of the association between a 1-unit increase in HbA1c and the primary cardiovascular outcomes.

© 2022 Htoo PT et al. *JAMA Network Open*.

eTable 1. Unmatched Distribution of Baseline Characteristics

**Characteristic**	**Empagliflozin**	**Liraglutide**		**Empagliflozin**	**Sitagliptin**	
	**N=35,721**	**N=49,400**	**ASD**	**N=25,285**	**N=198,893**	**ASD**
Age, years (Mean, SD)	72.4(5.34)	71.4(4.87)	0.193	71.9(5.03)	74.2(6.60)	0.388
Age categories						
66-69	12,903 (36.1%)	21,377 (43.3%)	0.147	9,898 (39.1%)	58,350 (29.3%)	0.208
70-74	12,164 (34.1%)	16,499 (33.4%)	0.014	8,744 (34.6%)	58,610 (29.5%)	0.11
75-79	6,719 (18.8%)	7,911 (16.0%)	0.074	4,448 (17.6%)	40,621 (20.4%)	0.072
80-84	2,740 (7.7%)	2,685 (5.4%)	0.09	1,590 (6.3%)	23,717 (11.9%)	0.197
≥85	1,195 (3.3%)	928 (1.9%)	0.092	605 (2.4%)	17,595 (8.8%)	0.283
Gender - Female	16,753 (46.9%)	27,793 (56.3%)	0.188	11,556 (45.7%)	111,998 (56.3%)	0.213
Geographic region						
Northeast	6,747 (18.9%)	7,719 (15.6%)	0.086	4,492 (17.8%)	36,958 (18.6%)	0.021
South	15,114 (42.3%)	21,515 (43.6%)	0.025	10,815 (42.8%)	84,458 (42.5%)	0.006
Midwest	6,648 (18.6%)	11,786 (23.9%)	0.129	4,930 (19.5%)	42,886 (21.6%)	0.051
West	7,212 (20.2%)	8,380 (17.0%)	0.083	5,048 (20.0%)	34,591 (17.4%)	0.066
Race categories						

© 2022 Htoo PT et al. *JAMA Network Open*.

African American/Black	2,508 (7.0%)	3,797 (7.7%)	0.025	1,649 (6.5%)	20,936 (10.5%)	0.144
Asian	1,452 (4.1%)	871 (1.8%)	0.137	679 (2.7%)	8,916 (4.5%)	0.097
Hispanic	1,123 (3.1%)	1,066 (2.2%)	0.061	630 (2.5%)	7,951 (4.0%)	0.085
White	28,681 (80.3%)	41,685 (84.4%)	0.107	21,083 (83.4%)	152,221 (76.5%)	0.172
Other or unknown[a]	1,957 (5.5%)	1,981 (4.0%)	0.069	1,244 (4.9%)	8,869 (4.5%)	0.022
Combined comorbidity score, Mean, (SD)	1.4(1.95)	1.7(2.15)	0.132	1.4(1.96)	1.5(2.25)	0.051
Frailty Score, Mean, (SD)	0.2(0.05)	0.2(0.06)	0.23	0.2(0.05)	0.2(0.06)	0.17
Frailty Score: (Categories)						
0.00 - 0.09	739 (2.1%)	555 (1.1%)	0.075	513 (2.0%)	3,234 (1.6%)	0.03
0.10 - 0.19	23,958 (67.1%)	29,238 (59.2%)	0.164	16,847 (66.6%)	121,336 (61.0%)	0.117
≥ 0.20	11,024 (30.9%)	19,607 (39.7%)	0.186	7,925 (31.3%)	74,323 (37.4%)	0.127
Overweight	3,962 (11.1%)	3,779 (7.6%)	0.118	2,665 (10.5%)	16,741 (8.4%)	0.073
Obesity	12,415 (34.8%)	22,892 (46.3%)	0.238	10,074 (39.8%)	52,895 (26.6%)	0.284
Smoking	7,886 (22.1%)	11,573 (23.4%)	0.032	5,912 (23.4%)	42,063 (21.1%)	0.054
Alcohol abuse or dependence	399 (1.1%)	515 (1.0%)	0.007	309 (1.2%)	2,485 (1.2%)	0.002

© 2022 Htoo PT et al. *JAMA Network Open*.

Drug abuse or dependence	596 (1.7%)	1,021 (2.1%)	0.029	428 (1.7%)	3,523 (1.8%)	0.006
Diabetic nephropathy	5,478 (15.3%)	10,300 (20.9%)	0.144	4,006 (15.8%)	29,580 (14.9%)	0.027
Diabetic retinopathy	4,953 (13.9%)	7,879 (15.9%)	0.059	3,711 (14.7%)	23,539 (11.8%)	0.084
Diabetes with other ophthalmic manifestations	3,635 (10.2%)	6,177 (12.5%)	0.073	2,445 (9.7%)	25,307 (12.7%)	0.097
Diabetic neuropathy	9,843 (27.6%)	15,928 (32.2%)	0.103	7,399 (29.3%)	48,179 (24.2%)	0.114
Diabetes with peripheral circulatory disorders	466 (1.3%)	1,749 (3.5%)	0.146	288 (1.1%)	7,619 (3.8%)	0.174
Diabetic foot	1,045 (2.9%)	2,011 (4.1%)	0.062	812 (3.2%)	6,201 (3.1%)	0.005
Infection of lower extremities (cellulitis or osteomyelitis)	1,941 (5.4%)	3,215 (6.5%)	0.045	1,384 (5.5%)	11,240 (5.7%)	0.008
Lower-limb amputations	201 (0.6%)	392 (0.8%)	0.028	165 (0.7%)	1,221 (0.6%)	0.005
Erectile dysfunction	1,788 (5.0%)	2,064 (4.2%)	0.04	1,363 (5.4%)	6,799 (3.4%)	0.096
Hypoglycemia	3,630 (10.2%)	5,292 (10.7%)	0.018	2,793 (11.0%)	17,486 (8.8%)	0.075
Hyperglycemia	17,441 (48.8%)	20,633 (41.8%)	0.142	12,776 (50.5%)	64,896 (32.6%)	0.369
Diabetic ketoacidosis	93 (0.3%)	198 (0.4%)	0.024	70 (0.3%)	799 (0.4%)	0.021
Hyperosmolar hyperglycemic nonketotic syndrome	347 (1.0%)	488 (1.0%)	0.002	262 (1.0%)	1,676 (0.8%)	0.02

© 2022 Htoo PT et al. *JAMA Network Open*.

Diabetes with other complications	4,106 (11.5%)	5,645 (11.4%)	0.002	3,023 (12.0%)	17,206 (8.7%)	0.109
Diabetes mellitus without mention of complications	33,606 (94.1%)	47,097 (95.3%)	0.056	23,711 (93.8%)	190,808 (95.9%)	0.098
Number of anti-DM medications on cohort entry, Mean, (SD)	1.6(0.96)	1.4(0.95)	0.14	1.4(0.91)	1.2(0.79)	0.282
No use of anti-DM medications in prior 365 days	1,355 (3.8%)	2,562 (5.2%)	0.067	1,213 (4.8%)	21,489 (10.8%)	0.225
Initiation of empagliflozin or comparator monotherapy	1,051 (2.9%)	2,328 (4.7%)	0.092	1,043 (4.1%)	14,137 (7.1%)	0.13
DUAL therapy with metformin	4,089 (11.4%)	4,158 (8.4%)	0.101	3,740 (14.8%)	32,553 (16.4%)	0.043
Metformin (any use)	28,427 (79.6%)	34,165 (69.2%)	0.24	19,881 (78.6%)	152,356 (76.6%)	0.049
Metformin (concurrent use)	22,663 (63.4%)	25,840 (52.3%)	0.227	16,011 (63.3%)	123,027 (61.9%)	0.03
Metformin (past use)	5,764 (16.1%)	8,325 (16.9%)	0.019	3,870 (15.3%)	29,329 (14.7%)	0.016
Sulfonylureas - 2nd generation (any use)	16,507 (46.2%)	21,987 (44.5%)	0.034	10,724 (42.4%)	93,931 (47.2%)	0.097

© 2022 Htoo PT et al. *JAMA Network Open*.

Sulfonylureas - 2nd generation (concurrent use)	12,603 (35.3%)	16,105 (32.6%)	0.057	8,167 (32.3%)	73,079 (36.7%)	0.094
Sulfonylureas - 2nd generation (past use)	3,904 (10.9%)	5,882 (11.9%)	0.031	2,557 (10.1%)	20,852 (10.5%)	0.012
Thiazolidinediones (any use)	3,935 (11.0%)	4,883 (9.9%)	0.037	2,727 (10.8%)	15,469 (7.8%)	0.104
Thiazolidinediones (concurrent use)	2,686 (7.5%)	3,255 (6.6%)	0.036	1,912 (7.6%)	10,258 (5.2%)	0.099
Thiazolidinediones (past use)	1,249 (3.5%)	1,628 (3.3%)	0.011	815 (3.2%)	5,211 (2.6%)	0.036
DPP4i (any use)	14,999 (42.0%)	14,459 (29.3%)	0.268	4,901 (19.4%)	6,442 (3.2%)	0.527
DPP4i (concurrent use)	11,343 (31.8%)	8,573 (17.4%)	0.339	3,222 (12.7%)	2,464 (1.2%)	0.463
DPP4i (past use)	3,656 (10.2%)	5,886 (11.9%)	0.054	1,679 (6.6%)	3,978 (2.0%)	0.23
Insulins (any use)	8,401 (23.5%)	21,635 (43.8%)	0.439	7,982 (31.6%)	30,931 (15.6%)	0.384
Insulins (concurrent use)	6,376 (17.8%)	16,648 (33.7%)	0.368	6,129 (24.2%)	22,017 (11.1%)	0.351
Insulins (past use)	2,025 (5.7%)	4,987 (10.1%)	0.165	1,853 (7.3%)	8,915 (4.5%)	0.121
Long term use of insulin	5,794 (16.2%)	13,041 (26.4%)	0.251	5,622 (22.2%)	20,314 (10.2%)	0.33
Miscellaneous anti-DM medications	1,114 (3.1%)	1,520 (3.1%)	0.002	631 (2.5%)	4,821 (2.4%)	0.005

© 2022 Htoo PT et al. *JAMA Network Open*.

Hypertension	32,773 (91.7%)	45,694 (92.5%)	0.028	23,254 (92.0%)	181,038 (91.0%)	0.034
Hyperlipidemia	30,997 (86.8%)	43,450 (88.0%)	0.036	21,942 (86.8%)	170,358 (85.7%)	0.033
Acute MI	987 (2.8%)	1,172 (2.4%)	0.025	724 (2.9%)	5,563 (2.8%)	0.004
MI sequelae/old MI	2,245 (6.3%)	3,000 (6.1%)	0.009	1,758 (7.0%)	11,491 (5.8%)	0.048
Stable angina	3,146 (8.8%)	3,406 (6.9%)	0.071	2,297 (9.1%)	12,605 (6.3%)	0.103
Acute coronary syndrome/ unstable angina	1,438 (4.0%)	1,628 (3.3%)	0.039	1,066 (4.2%)	6,368 (3.2%)	0.054
Coronary atherosclerosis	12,981 (36.3%)	16,726 (33.9%)	0.052	9,590 (37.9%)	63,054 (31.7%)	0.131
Coronary procedure	1,158 (3.2%)	1,129 (2.3%)	0.058	918 (3.6%)	4,549 (2.3%)	0.079
History of coronary procedure	5,202 (14.6%)	6,359 (12.9%)	0.049	3,951 (15.6%)	22,959 (11.5%)	0.119
Congestive heart failure	4,514 (12.6%)	7,395 (15.0%)	0.068	3,389 (13.4%)	29,429 (14.8%)	0.04
Cardiomyopathy	1,594 (4.5%)	2,329 (4.7%)	0.012	1,213 (4.8%)	9,340 (4.7%)	0.005
Atrial fibrillation	4,698 (13.2%)	6,308 (12.8%)	0.011	3,504 (13.9%)	27,635 (13.9%)	0.001
Cardiac conduction disorders	2,385 (6.7%)	3,234 (6.5%)	0.005	1,736 (6.9%)	13,407 (6.7%)	0.005
Other cardiac dysrhythmia	5,275 (14.8%)	7,042 (14.3%)	0.015	3,820 (15.1%)	31,513 (15.8%)	0.02
Valve disorders	5,236 (14.7%)	6,713 (13.6%)	0.031	3,707 (14.7%)	29,620 (14.9%)	0.007

© 2022 Htoo PT et al. *JAMA Network Open*.

Other cardiovascular disease	6,570 (18.4%)	8,025 (16.2%)	0.057	4,915 (19.4%)	30,947 (15.6%)	0.102
Ischemic stroke	4,582 (12.8%)	5,963 (12.1%)	0.023	3,227 (12.8%)	26,047 (13.1%)	0.01
Transient ischemic attack	983 (2.8%)	1,350 (2.7%)	0.001	677 (2.7%)	6,557 (3.3%)	0.036
Other cerebrovascular conditions	2,711 (7.6%)	4,048 (8.2%)	0.022	1,976 (7.8%)	19,347 (9.7%)	0.068
PAD and generalized/unspecified atherosclerosis	4,948 (13.9%)	7,153 (14.5%)	0.018	3,420 (13.5%)	29,521 (14.8%)	0.038
Acute Kidney Injury	1,267 (3.5%)	3,073 (6.2%)	0.124	877 (3.5%)	13,748 (6.9%)	0.156
Chronic kidney disease	5,736 (16.1%)	13,074 (26.5%)	0.256	4,040 (16.0%)	43,471 (21.9%)	0.151
Chronic kidney disease Stage 1-2	1,661 (4.6%)	2,615 (5.3%)	0.03	1,176 (4.7%)	9,039 (4.5%)	0.005
Chronic kidney disease Stage 3-4	3,729 (10.4%)	9,996 (20.2%)	0.274	2,616 (10.3%)	31,070 (15.6%)	0.157
Chronic kidney disease unspecified	1,963 (5.5%)	5,528 (11.2%)	0.207	1,392 (5.5%)	18,661 (9.4%)	0.148
Hypertensive nephropathy	2,776 (7.8%)	6,801 (13.8%)	0.194	1,962 (7.8%)	22,936 (11.5%)	0.128
Proteinuria	2,083 (5.8%)	3,858 (7.8%)	0.079	1,570 (6.2%)	10,801 (5.4%)	0.033

© 2022 Htoo PT et al. *JAMA Network Open*.

Miscellaneous renal disease	3,498 (9.8%)	7,072 (14.3%)	0.139	2,488 (9.8%)	26,206 (13.2%)	0.105
Urinary tract infection	5,233 (14.6%)	9,144 (18.5%)	0.104	3,525 (13.9%)	38,472 (19.3%)	0.145
Kidney and urinary stone	1,731 (4.8%)	2,576 (5.2%)	0.017	1,228 (4.9%)	9,054 (4.6%)	0.014
Disorders of electrolyte	2,510 (7.0%)	4,662 (9.4%)	0.088	1,704 (6.7%)	20,569 (10.3%)	0.129
Disorders of fluid balance	1,227 (3.4%)	2,133 (4.3%)	0.046	842 (3.3%)	10,444 (5.3%)	0.095
Edema	4,530 (12.7%)	8,494 (17.2%)	0.127	3,354 (13.3%)	28,501 (14.3%)	0.031
COPD	4,442 (12.4%)	7,222 (14.6%)	0.064	3,143 (12.4%)	29,206 (14.7%)	0.066
Asthma	3,253 (9.1%)	5,457 (11.0%)	0.064	2,355 (9.3%)	18,789 (9.4%)	0.005
Obstructive sleep apnea	6,129 (17.2%)	11,910 (24.1%)	0.172	5,161 (20.4%)	26,007 (13.1%)	0.197
Pneumonia	1,682 (4.7%)	2,664 (5.4%)	0.031	1,221 (4.8%)	12,066 (6.1%)	0.055
Osteoarthritis	10,818 (30.3%)	16,119 (32.6%)	0.051	7,798 (30.8%)	58,638 (29.5%)	0.03
Osteoporosis without fractures	2,639 (7.4%)	3,611 (7.3%)	0.003	1,662 (6.6%)	17,671 (8.9%)	0.087
Fractures	743 (2.1%)	1,215 (2.5%)	0.025	536 (2.1%)	4,459 (2.2%)	0.008
Falls	1,836 (5.1%)	2,791 (5.6%)	0.023	1,298 (5.1%)	11,933 (6.0%)	0.038
Hypothyroidism	8,915 (25.0%)	13,549 (27.4%)	0.056	6,314 (25.0%)	49,778 (25.0%)	0.001

© 2022 Htoo PT et al. *JAMA Network Open*.

Hyperthyroidism and other thyroid gland disorders	3,490 (9.8%)	4,669 (9.5%)	0.011	2,582 (10.2%)	15,173 (7.6%)	0.091
Non-alcoholic steatohepatitis/ fatty liver disease	1,995 (5.6%)	2,761 (5.6%)	0	1,502 (5.9%)	8,352 (4.2%)	0.079
Liver disease	1,546 (4.3%)	1,966 (4.0%)	0.017	1,070 (4.2%)	8,349 (4.2%)	0.002
Depression	5,147 (14.4%)	9,799 (19.8%)	0.144	3,903 (15.4%)	31,453 (15.8%)	0.01
Anxiety and sleep disorders	7,317 (20.5%)	11,651 (23.6%)	0.075	5,345 (21.1%)	40,584 (20.4%)	0.018
Dementia	1,637 (4.6%)	2,048 (4.1%)	0.021	1,036 (4.1%)	15,087 (7.6%)	0.149
Psychosis	249 (0.7%)	463 (0.9%)	0.027	152 (0.6%)	3,458 (1.7%)	0.106
Delirium	357 (1.0%)	523 (1.1%)	0.006	237 (0.9%)	3,247 (1.6%)	0.062
ACEi and ARBs	28,377 (79.4%)	39,703 (80.4%)	0.023	20,047 (79.3%)	153,612 (77.2%)	0.05
Beta blockers	17,862 (50.0%)	25,087 (50.8%)	0.016	12,840 (50.8%)	96,207 (48.4%)	0.048
Calcium channel blockers	12,183 (34.1%)	17,281 (35.0%)	0.018	8,435 (33.4%)	72,879 (36.6%)	0.069
Nitrates and other antianginal agents	3,728 (10.4%)	5,106 (10.3%)	0.003	2,678 (10.6%)	19,544 (9.8%)	0.025
Thiazide and thiazide-like diuretics	5,509 (15.4%)	8,780 (17.8%)	0.063	4,135 (16.4%)	32,402 (16.3%)	0.002

© 2022 Htoo PT et al. *JAMA Network Open*.

Loop diuretics	6,010 (16.8%)	12,687 (25.7%)	0.218	4,676 (18.5%)	41,271 (20.8%)	0.057
MRA (potassium-sparing diuretics)	1,757 (4.9%)	3,176 (6.4%)	0.065	1,375 (5.4%)	9,627 (4.8%)	0.027
Other potassium -sparing diuretics and antihypertensive medications	3,456 (9.7%)	6,078 (12.3%)	0.084	2,442 (9.7%)	23,516 (11.8%)	0.07
Digoxin	904 (2.5%)	1,122 (2.3%)	0.017	633 (2.5%)	6,195 (3.1%)	0.037
Entresto	182 (0.5%)	101 (0.2%)	0.051	140 (0.6%)	326 (0.2%)	0.065
Antiarrhythmics	893 (2.5%)	1,312 (2.7%)	0.01	680 (2.7%)	5,382 (2.7%)	0.001
Anticoagulants (oral)	3,763 (10.5%)	5,476 (11.1%)	0.018	2,782 (11.0%)	22,140 (11.1%)	0.004
Anticoagulants (injectables)	190 (0.5%)	389 (0.8%)	0.032	163 (0.6%)	1,428 (0.7%)	0.009
Antiplatelet agents	5,537 (15.5%)	6,850 (13.9%)	0.046	3,990 (15.8%)	27,455 (13.8%)	0.056
Statins	28,925 (81.0%)	39,299 (79.6%)	0.036	20,465 (80.9%)	151,259 (76.1%)	0.119
PCSK9 inhibitors and other-lipid lowering agents	6,737 (18.9%)	9,278 (18.8%)	0.002	4,693 (18.6%)	31,124 (15.6%)	0.077
COPD & asthma medications	7,153 (20.0%)	11,358 (23.0%)	0.072	5,079 (20.1%)	39,632 (19.9%)	0.004

© 2022 Htoo PT et al. *JAMA Network Open*.

Corticosteroids (oral)	6,211 (17.4%)	9,441 (19.1%)	0.045	4,420 (17.5%)	35,229 (17.7%)	0.006
Antiosteoporosis agents	1,698 (4.8%)	2,142 (4.3%)	0.02	977 (3.9%)	11,564 (5.8%)	0.091
NSAIDs	8,881 (24.9%)	12,822 (26.0%)	0.025	6,151 (24.3%)	49,159 (24.7%)	0.009
Opioids	11,480 (32.1%)	19,949 (40.4%)	0.172	8,501 (33.6%)	68,639 (34.5%)	0.019
Gabapentinoids	6,196 (17.3%)	11,206 (22.7%)	0.134	4,586 (18.1%)	35,707 (18.0%)	0.005
UTI antibiotics	8,040 (22.5%)	13,581 (27.5%)	0.115	5,622 (22.2%)	52,304 (26.3%)	0.095
Antidepressants	9,614 (26.9%)	17,841 (36.1%)	0.199	7,300 (28.9%)	55,988 (28.1%)	0.016
Anxiolytics/ hypnotics	2,671 (7.5%)	4,289 (8.7%)	0.044	1,912 (7.6%)	14,398 (7.2%)	0.012
Benzodiazepines	4,786 (13.4%)	7,637 (15.5%)	0.059	3,458 (13.7%)	28,585 (14.4%)	0.02
Antipsychotics	776 (2.2%)	1,296 (2.6%)	0.029	542 (2.1%)	6,514 (3.3%)	0.07
Antiparkinsonian medications	1,149 (3.2%)	2,403 (4.9%)	0.084	836 (3.3%)	7,366 (3.7%)	0.022
Dementia medications	987 (2.8%)	1,207 (2.4%)	0.02	565 (2.2%)	9,643 (4.8%)	0.142
Internist (-365 days to CED)	31,975 (89.5%)	44,006 (89.1%)	0.014	22,650 (89.6%)	178,324 (89.7%)	0.003
Internist (-30 days to CED)	22,375 (62.6%)	29,402 (59.5%)	0.064	15,418 (61.0%)	133,532 (67.1%)	0.129
Internist (-365 days to -31 days before CED)	31,211 (87.4%)	43,006 (87.1%)	0.01	22,089 (87.4%)	171,575 (86.3%)	0.032

© 2022 Htoo PT et al. *JAMA Network Open*.

Internist (Number of visits), mean, (SD)	15.2(18.25)	15.8(20.11)	0.03	14.8(18.20)	15.5(18.68)	0.04
Endocrinologist (-365 days to CED)	7,877 (22.1%)	13,474 (27.3%)	0.121	6,569 (26.0%)	24,636 (12.4%)	0.35
Endocrinologist (-30 days to CED)	5,706 (16.0%)	9,439 (19.1%)	0.082	4,818 (19.1%)	15,372 (7.7%)	0.337
Endocrinologist (-365 days to -31 days before CED)	6,329 (17.7%)	10,360 (21.0%)	0.082	5,404 (21.4%)	18,964 (9.5%)	0.332
Endocrinologist (Number of visits), mean, (SD)	2.0(7.35)	2.3(7.28)	0.042	2.4(8.08)	1.0(4.81)	0.216
Cardiologist (-365 days to CED)	18,190 (50.9%)	24,446 (49.5%)	0.029	13,244 (52.4%)	94,034 (47.3%)	0.102
Cardiologist (-30 days to CED)	5,999 (16.8%)	6,457 (13.1%)	0.105	4,405 (17.4%)	30,628 (15.4%)	0.055
Cardiologist (-365 days to -31 days before CED)	17,244 (48.3%)	23,330 (47.2%)	0.021	12,573 (49.7%)	87,110 (43.8%)	0.119
Cardiologist (Number of visits), mean, (SD)	4.6(9.56)	4.1(9.13)	0.05	4.8(9.67)	4.1(9.10)	0.072
Nephrologist (-365 days to CED)	1,739 (4.9%)	5,507 (11.1%)	0.233	1,222 (4.8%)	16,207 (8.1%)	0.135

© 2022 Htoo PT et al. *JAMA Network Open*.

Nephrologist (-30 days to CED)	440 (1.2%)	1,391 (2.8%)	0.113	331 (1.3%)	5,827 (2.9%)	0.113
Nephrologist (-365 days to -31 days before CED)	1,632 (4.6%)	5,208 (10.5%)	0.227	1,139 (4.5%)	13,964 (7.0%)	0.108
Nephrologist (Number of visits), mean, (SD)	0.3(2.31)	0.6(3.15)	0.121	0.3(2.41)	0.5(3.01)	0.072
Electrocardiogram	18,863 (52.8%)	25,299 (51.2%)	0.032	13,419 (53.1%)	102,299 (51.4%)	0.033
Electrocardiogram (Number of tests), mean, (SD)	1.5(2.39)	1.4(2.47)	0.006	1.5(2.46)	1.5(2.61)	0.008
ECG & Other cardiac imaging	10,995 (30.8%)	14,958 (30.3%)	0.011	7,926 (31.3%)	58,455 (29.4%)	0.043
ECG & Other cardiac imaging (Number of tests), mean, (SD)	0.8(1.57)	0.8(1.54)	0.009	0.8(1.63)	0.7(1.52)	0.057
Cardiovascular stress test	5,454 (15.3%)	7,390 (15.0%)	0.009	4,001 (15.8%)	25,046 (12.6%)	0.093
HbA1c test order	34,726 (97.2%)	47,912 (97.0%)	0.014	24,641 (97.5%)	190,117 (95.6%)	0.102
HbA1c test order (Number of tests), mean, (SD)	2.8(1.32)	2.9(1.36)	0.045	2.9(1.31)	2.6(1.34)	0.177
Glucose test and monitoring	12,345 (34.6%)	18,568 (37.6%)	0.063	8,922 (35.3%)	64,557 (32.5%)	0.06

© 2022 Htoo PT et al. *JAMA Network Open*.

Glucose test and monitoring (Number of tests), mean, (SD)	0.9(1.85)	1.0(1.95)	0.045	0.9(1.90)	0.8(2.27)	0.049
Microalbuminuria/proteinuria test order	23,247 (65.1%)	32,539 (65.9%)	0.017	16,673 (65.9%)	116,088 (58.4%)	0.157
Microalbuminuria/proteinuria test order (Number of tests), mean, (SD)	1.0(1.06)	1.1(1.14)	0.056	1.0(1.06)	0.9(1.04)	0.125
Metabolic or renal/creatinine panel test order	34,885 (97.7%)	48,137 (97.4%)	0.014	24,689 (97.6%)	192,777 (96.9%)	0.044
Metabolic or renal/creatinine panel test order (Number of tests), mean, (SD)	3.4(2.23)	3.6(2.57)	0.118	3.4(2.25)	3.4(2.46)	0.008
Lipid test order	32,873 (92.0%)	44,685 (90.5%)	0.056	23,242 (91.9%)	177,378 (89.2%)	0.094
Lipid test order (Number of tests), mean, (SD)	2.2(1.55)	2.1(1.58)	0.053	2.1(1.58)	2.0(1.50)	0.109
Uric acid test order	5,027 (14.1%)	7,482 (15.1%)	0.03	3,482 (13.8%)	27,476 (13.8%)	0.001
Vitamin D test order	11,536 (32.3%)	16,783 (34.0%)	0.036	8,022 (31.7%)	61,213 (30.8%)	0.02
PTH test order	2,086 (5.8%)	5,269 (10.7%)	0.176	1,538 (6.1%)	13,944 (7.0%)	0.038

© 2022 Htoo PT et al. *JAMA Network Open*.

Hospitalization event (Number of), mean, (SD)	0.2(0.53)	0.2(0.57)	0.051	0.2(0.54)	0.3(0.69)	0.131
LOS (-365 days to -31 days before CED), mean, (SD)	0.8(3.72)	0.9(3.62)	0.04	0.8(3.27)	1.0(4.73)	0.062
LOS (-30 days to CED), mean, (SD)	0.1(0.99)	0.1(0.92)	0.002	0.1(0.98)	0.5(2.41)	0.192
ED event (Number of), mean, (SD)	0.7(1.66)	0.8(1.89)	0.075	0.7(1.73)	0.9(2.08)	0.108
Distinct medications ALL, mean, (SD)	13.2(5.77)	14.8(6.11)	0.262	13.3(5.75)	12.8(5.87)	0.098
Distinct medications all BRAND, mean, (SD)	3.1(1.95)	3.6(2.17)	0.215	3.0(1.96)	2.7(1.90)	0.17
Cost Total ($), mean, (SD)	11835.8(14020.42)	13441.0(16301.11)	0.106	12513.0(32756.46)	10769.7(15528.41)	0.068
Cost inpatient ($), mean, (SD)	2075.3(7897.58)	2238.2(7952.80)	0.021	2177.9(8119.74)	2884.4(9725.25)	0.079
Cost outpatient ($), mean, (SD)	1937.3(4627.23)	2329.2(7898.34)	0.061	2112.4(5230.43)	1863.9(4294.26)	0.052
Cost pharmacy for DM medications ($), mean, (SD)	3307.0(4102.80)	3928.6(4413.29)	0.146	3588.9(5267.34)	1355.5(2287.93)	0.55

© 2022 Htoo PT et al. *JAMA Network Open*.

Cost pharmacy for non-DM medications ($), mean, (SD)	4381.4(6583.45)	3664.3(7154.91)	0.104	3597.3(29226.04)	3152.7(7068.80)	0.021
Cost pharmacy ($), mean, (SD)	905.3(943.58)	982.4(1001.04)	0.079	989.8(1766.09)	587.5(729.96)	0.298
Cost home healthcare ($), mean, (SD)	322.3(1743.58)	448.0(2186.89)	0.064	288.7(1591.43)	590.5(2368.16)	0.15
Flu vaccine	22,607 (63.3%)	31,825 (64.4%)	0.024	16,256 (64.3%)	118,837 (59.7%)	0.094
Pneumoccocal vaccine	7,956 (22.3%)	11,641 (23.6%)	0.031	5,747 (22.7%)	41,225 (20.7%)	0.049
Breast mammography or magnetic resonance imaging	7,968 (22.3%)	13,538 (27.4%)	0.118	5,651 (22.3%)	45,268 (22.8%)	0.01
Prostate examination or PSA	10,433 (29.2%)	11,082 (22.4%)	0.155	7,420 (29.3%)	44,367 (22.3%)	0.161
Colonoscopy	3,708 (10.4%)	5,359 (10.8%)	0.015	2,839 (11.2%)	18,179 (9.1%)	0.069
Pap smear	1,703 (4.8%)	2,801 (5.7%)	0.041	1,195 (4.7%)	8,953 (4.5%)	0.011
Bone mineral density	2,986 (8.4%)	4,577 (9.3%)	0.032	2,043 (8.1%)	16,408 (8.2%)	0.006

ACEI: angiotensin converting enzyme inhibitors; ARB: angiotensin receptor blockers; BB: betab lockers; CCB: calcium channel blockers; CED: cohort entry date; COPD: chronic obstructive pulmonary diseases; DM: diabetes mellitus; DPP4i: dipeptidyl peptidase-4 inhibitors; MRA: mineralocorticoid receptor antagonists; PAD: peripheral arterial diseases; PSA: prostate surface antigen; SD: standard deviation; ASD: standardized mean differences;

[a] Includes American Indian/Alaskan Native or unknown.

© 2022 Htoo PT et al. *JAMA Network Open*.

eTable 2. Follow-up Time and Censoring Reason for Primary Outcomes Between 1:1 PS-Matched Initiators of Empagliflozin vs Liraglutide or Sitagliptin[a]

**Overall population**	**Overall N = 45,788**	**Empagliflozin N = 22,894**	**Liraglutide N = 22,894**
**Modified MACE**			
Follow-up, mean (SD), days	242 (227)	250 (232)	233 (221)
Follow-up, median (25th, 75th IQR), days	153 [88, 308]	163 [88, 324]	148 [88, 292]
Censoring reasons (%):			
Treatment discontinuation	48.8	46.6	51.0
End of study - 12/31/2018	36.2	37.3	31.0
Treatment switching	8.3	9.4	7.4
Disenrollment	4.6	4.7	4.4
Composite CV outcome	2.0	2.0	2.0
All-cause mortality	0	0	0
**HHF outcome**			
Follow-up, mean (SD), days	242 (227)	250 (232)	233 (221)
Follow-up, median (25th, 75th IQR), days	157 [88, 321]	157 [88, 315]	157 [88, 327]
Censoring reasons (%):			
Treatment discontinuation	49.1	46.9	51.2
End of study - 12/31/2018	36.4	37.5	35.3
Treatment switching	8.4	9.4	7.4
Disenrollment	4.6	4.8	4.5
HHF outcome	0.7	0.6	0.8
All-cause mortality	0.8	0.8	0.8
**Overall population**	**Overall N = 45,624**	**Empagliflozin N = 22,812**	**Sitagliptin N = 22,812**
**Modified MACE**			
Follow-up, mean (SD), days	234 (210)	229 (207)	239 (212)
Follow-up, median (25th, 75th IQR), days	155 [88, 312]	151 [88, 302]	158 [88, 322]
Censoring reasons (%):			
Treatment discontinuation	42.4	42.1	42.8
End of study - 12/31/2018	44.1	43.1	45.0
Treatment switching	7.0	8.5	5.7
Disenrollment	4.3	4.0	4.6
Composite CV outcome	2.1	1.7	2.5
All-cause mortality	0	0	0
**HHF outcome**			
Follow-up, mean (SD), days	234 (210)	229 (207)	239 (212)

© 2022 Htoo PT et al. *JAMA Network Open*.

Follow-up, median (25th, 75th IQR), days	155 [88, 313]	151 [88, 303]	159 [88, 322]
Censoring reasons (%):			
Treatment discontinuation	42.6	43.1	42.2
End of study - 12/31/2018	44.3	43.3	45.3
Treatment switching	7.1	8.5	5.8
Disenrollment	4.3	4.0	4.6
HHF outcome development	0.8	0.5	1.1
All-cause mortality	0.8	0.7	1.0

PS: propensity-score; MACE: major adverse cardiovascular events; SD: standard deviation; IQR: interquartile range; HHF: hospitalization for heart failure

[a] Censoring was outcome-specific. The table reports follow-up and censoring information specific to the primary outcomes. Censoring reasons specific to other study outcomes may include minor variations.

© 2022 Htoo PT et al. *JAMA Network Open*.

eTable 3. Sensitivity Analyses for 1:1 PS-Matched Initiators of Empagliflozin vs Liraglutide or Sitagliptin

			Empaglilfozin	Comparator	Empaglilfozin vs. Comparator	
**Sensitivity analyses**	**Comparison**	**Primary outcomes**	**N events (IR/1000 PY)**	**N events (IR/1000 PY)**	**HR (95% CI)**	**RD/1000PY (95% CI)**
Metformin at baseline	Empagliflozin vs. Liraglutide	Modified MACE outcome[a]	314 (17680)	301 (17680)	0.97 (0.83, 1.14)	-0.53 (-4.64, 3.56)
		Hospitalization for heart failure (specific)	81 (17680)	131 (17680)	0.56 (0.43, 0.75)	-4.77 (-7.23, -2.38)
	Empagliflozin vs. Sitagliptin	Modified MACE outcome[a]	277 (18266)	417 (18266)	0.68 (0.59, 0.79)	-11.03 (-15.46, -6.64)
		Hospitalization for heart failure (specific)	74 (18266)	167 (18266)	0.46 (0.35, 0.60)	-7.60 (-10.22, -5.05)
No insulin at baseline	Empagliflozin vs. Liraglutide	Modified MACE outcome[a]	242 (13882)	224 (13882)	0.98 (0.82, 1.17)	-0.27 (-4.72, 4.16)
		Hospitalization for heart failure (specific)	62 (13882)	90 (13882)	0.61 (0.44, 0.85)	-3.64 (-6.25, -1.12)
	Empagliflozin vs. Sitagliptin	Modified MACE outcome[a]	226 (15634)	321 (15634)	0.74 (0.62, 0.88)	-8.01 (-12.60, -3.44)
		Hospitalization for heart failure (specific)	47 (15634)	130 (15634)	0.38 (0.27, 0.53)	-7.82 (-10.46, -5.29)

© 2022 Htoo PT et al. *JAMA Network Open*.

ITT 730 days	Empagliflozin vs. Liraglutide	Modified MACE outcome[a]	911 (22906)	949 (22906)	0.95 (0.87, 1.05)	-1.64 (-4.92, 1.63)
		Hospitalization for heart failure (specific)	285 (22906)	403 (22906)	0.70 (0.60, 0.82)	-4.64 (-6.63, -2.66)
	Empagliflozin vs. Sitagliptin	Modified MACE outcome[a]	774 (22822)	914 (22822)	0.83 (0.76, 0.92)	-6.62 (-10.15, -3.10)
		Hospitalization for heart failure (specific)	245 (22822)	394 (22822)	0.61 (0.52, 0.72)	-6.70 (-8.88, -4.55)
Exposure risk period 30 days	Empagliflozin vs. Liraglutide	Modified MACE outcome[a]	358 (22894)	319 (22894)	0.97 (0.83, 1.12)	-0.94 (-5.23, 3.31)
		Hospitalization for heart failure (specific)	89 (22894)	138 (22894)	0.54 (0.42, 0.71)	-5.53 (-8.10, -3.05)
	Empagliflozin vs. Sitagliptin	Modified MACE outcome[a]	302 (22812)	466 (22812)	0.67 (0.58, 0.78)	-11.91 (-16.34, -7.52)
		Hospitalization for heart failure (specific)	83 (22812)	221 (22812)	0.39 (0.30, 0.50)	-10.65 (-13.45, -7.93)

PS: propensity score; IR: Incidence rate; PY: person-years; HR: hazard ratio; CI: confidence intervals; RD: rate difference.

[a] Hospitalization for myocardial infarction, or ischemic or hemorrhagic stroke, all-cause mortality.

© 2022 Htoo PT et al. *JAMA Network Open*.

eTable 4. Number of Events, Incidence Rates, and Treatment Effect Estimates for 1:1 PS-Matched Initiators of Empagliflozin vs GLP-1RA or DPP-4 Inhibitor in the Overall Population

	**Empagliflozin**	**Comparator**	**Empagliflozin vs. comparator**	
**Primary outcomes**	**N events (IR/1,000 PY)**	**N events (IR/1,000 PY)**	**HR (95% CI)**	**RD/1,000PY (95% CI)**
**Empagliflozin vs. GLP-1RA (N of matched pairs = 31,716)**				
Modified MACE outcome[a]	582 (28.0)	577 (29.6)	0.94 (0.84, 1.06)	-1.55 (-4.88, 1.76)
Hospitalization for heart failure (specific)	161 (7.7)	225 (11.5)	0.66 (0.54, 0.81)	-3.79 (-5.73, -1.88)
Hospitalization for heart failure (broad)	794 (38.6)	906 (47.0)	0.82 (0.74, 0.90)	-8.46 (-12.54, -4.40)
**Empagliflozin vs. DPP-4i (N of matched pairs = 23,309)**				
Modified MACE outcome[a]	388 (26.6)	581 (38.8)	0.68 (0.60, 0.78)	-12.21 (-16.34, -8.10)
Hospitalization for heart failure (specific)	119 (8.1)	238 (15.8)	0.51 (0.41, 0.64)	-7.71 (-10.23, -5.24)
Hospitalization for heart failure (broad)	586 (40.5)	861 (58.3)	0.69 (0.62, 0.77)	-17.85 (-22.96, -12.77)

PS: propensity score; GLP-1RA: glucagon-like peptide-1 receptor agonists; DPP-4i: dipeptidyl peptidase-4 inhibitors; IR: Incidence rate; PY: person-years; HR: hazard ratio; CI: confidence intervals; RD: rate difference; CVD: cardiovascular disease.

[a] Hospitalization for myocardial infarction, or ischemic or hemorrhagic stroke, all-cause mortality.

© 2022 Htoo PT et al. *JAMA Network Open*.
